# Efficient activity of uracil DNA glycosylase (UNG2) in proliferating cells requires binding to proliferating cell nuclear antigen (PCNA) and replication protein A (RPA)

**DOI:** 10.1016/j.dnarep.2025.103918

**Published:** 2025-12-26

**Authors:** Rashmi S. Kulkarni, Brian P. Weiser

**Affiliations:** a Department of Cell & Molecular Biology, Rowan-Virtua School of Osteopathic Medicine, Rowan University, Stratford, NJ 08084, United States; b Department of Cell & Molecular Biology, Rowan-Virtua School of Translational Biomedical Engineering & Sciences, Rowan University, Stratford, NJ 08084, United States

**Keywords:** Base excision repair, Uracil DNA glycosylase, Replication protein A, PCNA, 5-fluorodeoxyuridine, Pemetrexed

## Abstract

The compounds pemetrexed and 5-fluorodeoxyuridine (FdU) are widely used for cancer therapies and disrupt cell proliferation by inducing DNA damage and stressing DNA replication. The drugs disrupt pyrimidine nucleotide metabolism and promote the accumulation of uracil bases in genomic DNA, which are repaired by uracil DNA glycosylase (UNG2) and downstream base excision repair proteins. UNG2 interacts with Proliferating Cell Nuclear Antigen (PCNA) and Replication Protein A (RPA), which localize to the replication fork during DNA damage responses to orchestrate DNA repair. In this work, we tested whether UNG2 requires interaction with PCNA and RPA to repair DNA damage in a colorectal cancer model during treatment with pemetrexed or FdU. We genetically knocked out UNG2 in HT29 cells and engineered the cells to express UNG2 variants that cannot bind to PCNA or RPA. We found that eliminating UNG2 activity or disrupting its interaction with PCNA or RPA sensitized the cells to the DNA-damaging effects of pemetrexed and FdU. The ability of UNG2 to localize to stalled replication forks was impaired when the enzyme could not interact with PCNA or RPA. Finally, disrupting the interaction of UNG2 with PCNA or RPA sensitized the cells to the cytotoxicity of the drugs. We concluded that certain cancers may be sensitized to pemetrexed and FdU by directly inhibiting the enzymatic activity of UNG2, by depleting UNG2 levels in the cell, or by impairing UNG2 function by inhibiting its protein-protein interactions.

## 1. Introduction

Lesions that accumulate in DNA stress mammalian DNA replication and can lead to cytotoxicity. This is a mechanism of action for chemotherapeutic agents that damage replicating DNA to the extent that it becomes irreparable, which triggers cell death in hyper-proliferative cancer cells. It follows that the efficacy of DNA damaging agents is counteracted by the activity of cellular DNA repair pathways, which are often dysregulated in cancer cells [1,2].

**Fig. 1.**
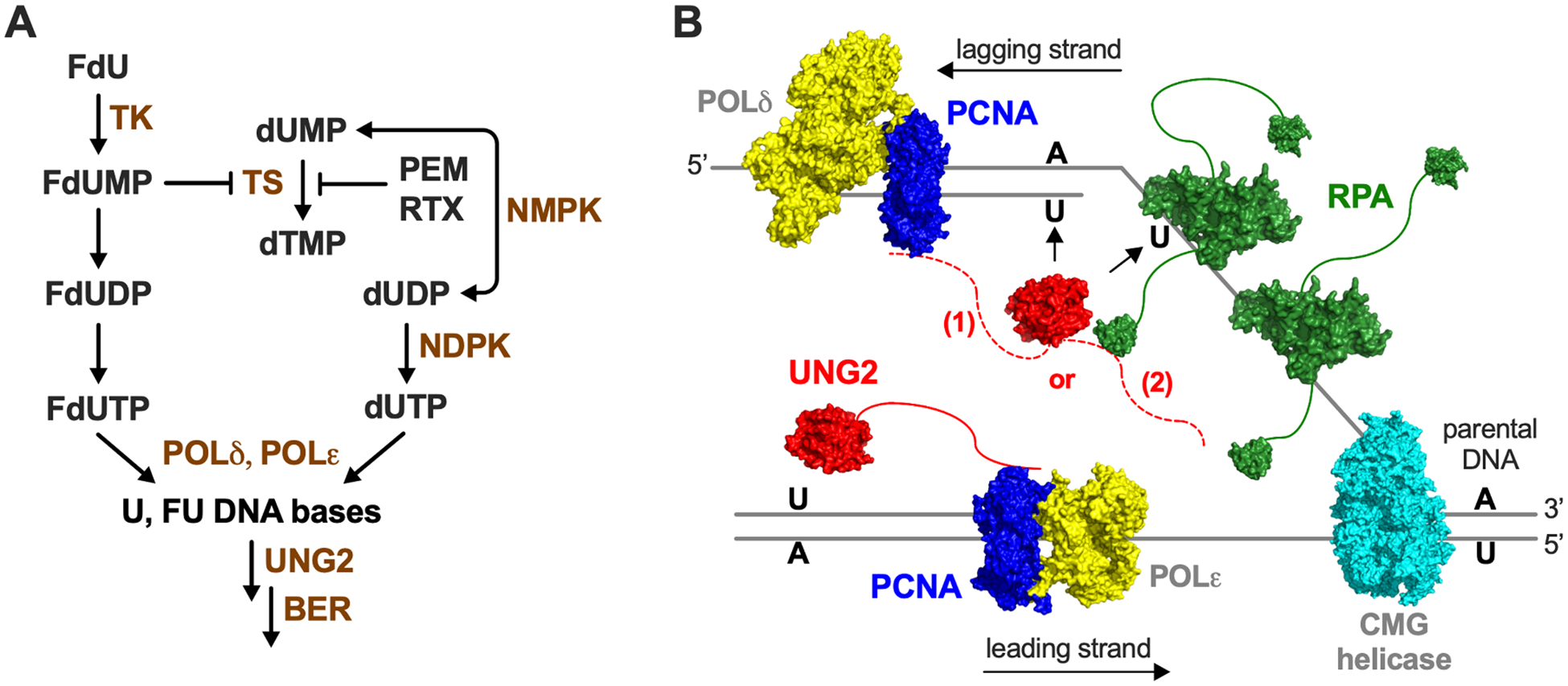
Pharmacology of TS inhibitors and a model of UNG2, PCNA, and RPA at the replication fork. (A) FdU is phosphorylated to yield FdUMP, a TS inhibitor. Further downstream, FdUMP is converted into FdUTP, which is used by replicative polymerases to incorporate 5-fluorouracil bases into DNA. Conversely, pemetrexed and raltitrexed directly inhibit TS and the production of deoxythymidine nucleotides. Instead, dUMP is ultimately metabolized into dUTP, which is used by polymerases to insert uracil bases into DNA. FdU also elevates uracil bases in DNA through TS inhibition. (B) At the replication fork, UNG2 interacts with PCNA to rapidly remove uracil bases from newly synthesized DNA. However, instead of interacting with PCNA (1), UNG2 can also interact with RPA (2) which binds naked ssDNA (the dashed lines indicate different orientations of the UNG2 N-terminal domain). RPA can target UNG2 to nearby dsDNA or ssDNA regions [16–18]. Uracil bases in DNA (or 5-fluorouracil bases) that are not excised by UNG2 can persist in genomic DNA until the next round of replication [13]. Panel A [4,5] and panel B [16,21,22] were adapted from other sources. BER, base excision repair; dTMP, deoxythymidine monophosphate; dUDP, deoxyuridine diphosphate; dUMP, deoxyuridine monophosphate; dUTP, deoxyuridine triphosphate; FdU, 5-fluorodeoxyuridine; FdUDP, 5-fluorodeoxyuridine diphosphate; FdUMP, 5-fluorodeoxyuridine monophosphate; FdUTP, 5-fluorodeoxyuridine triphosphate; FU, 5-fluorouracil; NDPK, nucleoside diphosphate kinase; NMPK, nucleoside monophosphate kinase; PEM, pemetrexed; POLδ, DNA polymerase δ; POL ε, DNA polymerase ε; RTX, raltitrexed; TK, thymidine kinase; TS, thymidylate synthase; U, uracil; UNG2, uracil DNA glycosylase 2.

This present work studies thymidylate synthase (TS) inhibitors, which are a class of drugs for colorectal and other cancers that promote the accumulation of uracil bases in genomic DNA, which are a source of stress for replicating cells. TS inhibitors such as pemetrexed and raltitrexed create an imbalance of pyrimidine nucleotide pools in the nucleus by depleting deoxythymidine triphosphate (dTTP) in favor of deoxyuridine triphosphate (dUTP), which is used by replicative polymerases to insert uracil bases into DNA (Fig. 1A). Additionally, the prodrug 5-fluorodeoxyuridine (FdU) is metabolized into a TS inhibitor, and FdU is also a source of unnatural 5-fluorouracil bases that are incorporated into DNA (Fig. 1A). Uracil DNA glycosylase (UNG2) is the primary enzyme responsible for removing uracil and 5-fluorouracil bases from genomic DNA, which it does by excising the base at the N-glycosidic bond [3,4]. UNG2 activity promotes resistance to FdU and TS inhibitors in many cancers because the enzyme initiates repair of the uracil lesions that the drugs induce [5]. Accordingly, inhibition or depletion of UNG2 may be a secondary strategy to improve the effectiveness of the drugs [5–7].

Previous studies with human cancer cells used genetic or functional knockout of UNG2 to demonstrate that the enzyme is critical for removing uracil and 5-fluoruracil bases from genomic DNA during FdU or TS inhibitor treatments, despite some variability in the extent to which different cell types rely on UNG2 [5,8]. Loss of UNG2 activity can result in significantly elevated levels of uracil bases in genomic DNA following treatment with pemetrexed and raltitrexed, and FdU elevates both uracil and 5-fluorouracil levels in DNA [5,9–12]. Increased levels of genomic uracil or 5-fluorouracil bases induce DNA damage phenotypes such as replication fork stalling or collapse, activation of DNA damage checkpoints or repair pathways, and increased susceptibility to strand breaks [5,9–11,13,14]. Finally, cells become hypersensitive to the cytotoxicity of pemetrexed, raltitrexed, and FdU when UNG2 is not available to avert significant DNA damage caused by the presence of uracil and 5-fluorouracil bases which triggers apoptosis [5,8–11,13].

To improve drug action, it is critical to understand the molecular mechanisms that UNG2 uses to repair DNA and counteract the effectiveness of FdU and TS inhibitors. UNG2 is a highly efficient enzyme that can bind DNA and quickly scan its bases to excise uracil and 5-fluorouracil lesions. While it is plausible that UNG2 scans bulk DNA in the nucleus, the enzyme becomes hindered when DNA packaging prevents access to the bases [15]. Instead, enzymology studies indicate that UNG2 can be targeted to specific lesion sites by other proteins which enhances its efficiency [16–18]. UNG2 contains a ~90 residue, largely disordered N-terminal domain that has binding sites for Proliferating Cell Nuclear Antigen (PCNA), which is the mammalian dsDNA binding clamp, as well as Replication Protein A (RPA), which is the primary ssDNA binding protein in the nucleus [19,20]. PCNA and RPA are critical for orchestrating most DNA replication and repair processes and localize to the replication fork, where they have been proposed to target UNG2 towards pre- or post-replicative uracil bases (Fig. 1B). In this work, we asked whether the interactions of UNG2 with PCNA or RPA were essential for the enzyme to efficiently excise uracil bases that are introduced into nuclear DNA. We found that UNG2 requires interaction with both PCNA and RPA to efficiently repair uracil lesions in the nucleus at the replication fork, and show that disrupting UNG2’s interaction with either protein significantly sensitizes cells to the DNA-damaging effects of pemetrexed and FdU.

## 2. Materials and methods

### 2.1. General cell culture

HT29 human colorectal cancer cells were purchased from ATCC and maintained at 37°C in an incubator with 5 % CO_2_ [23]. Unless otherwise stated, the HT29 cells and various stable cell lines derived from them were grown in DMEM (Corning #10–013-CV) supplemented with 10 % fetal bovine serum, 100 U/ml penicillin, and 100 μg/ml streptomycin (“full DMEM”).

### 2.2. Antibodies

The primary antibody for UNG2 detection by western blot and immunofluorescence was previously characterized [23–25] and was a generous gift from Dr. Salvatore J. Caradonna (Rowan University). The epitope(s) for this polyclonal rabbit antibody resides in the first 24 amino acids of full-length UNG2. For western blot, the UNG2 antibody was used with an HRP-conjugated goat anti-rabbit secondary antibody (Invitrogen catalog #65–6120), and for immunofluorescence we used an anti-rabbit secondary antibody that was conjugated to Alexa Fluor 594 (Invitrogen catalog #A11012). For detecting PCNA by immunofluorescence, we used a mouse monoclonal antibody from Cell Signaling Technology (catalog #2586) with an anti-mouse secondary antibody conjugated to Alexa Fluor 488 (Invitrogen catalog #A-11001). RPA was detected by immunofluorescence with a rat monoclonal antibody from Cell Signaling Technology that targets RPA32 (catalog #2208) and an anti-rat secondary antibody conjugated to Alexa Fluor 647 (Invitrogen catalog #A-21247). For detecting 8xHis-SUMO-UdgX by dot blot, we used a mouse monoclonal antibody directed toward a 6xHis tag (Invitrogen catalog #MA1–21315) with an HRP-conjugated goat anti-mouse secondary antibody (Invitrogen catalog #62–6520).

### 2.3. Expression of recombinant UNG2 proteins and fluorescence anisotropy assays

Recombinant UNG2(FF/AA) and recombinant UNG2(NR/DG) were produced from a pET21a plasmid in a manner identical to wild-type UNG2 [26] except that point mutations were first introduced into the gene with standard QuikChange/site directed mutagenesis. Expression and purification of 6xHis-PCNA was previously reported by our lab [26]. Recombinant RPA32 winged-helix domain (RPA32-WH) (amino acids 202–270) was expressed in BL21(DE3)pLysS cells from an addgene plasmid (#109795) that encoded an N-terminal 6xHis tag followed by a GST domain then the RPA32 domain [27]. The tagged protein was purified by standard Ni^2+^ column chromatography followed by size exclusion chromatography. All protein expression plasmids were verified by Sanger sequencing.

The recombinant proteins were used in fluorescence anisotropy binding assays that were previously reported [17,26,28,29]. Briefly, the interaction of UNG2 variants with PCNA was measured by a competition assay where a fluorescein-labeled peptide called Pogo-Ligase was displaced from its shared PCNA binding site by UNG2 [26,28,29]. Conversely, the interaction of UNG2 variants with the RPA32-WH was measured by a competition assay where a fluorescein-labeled peptide from SMARCAL1 was displaced from its conserved RPA32 binding site by UNG2 [17,26]. Successful displacement of peptides by UNG2 yielded sigmoidal dose-response datasets that were fit with curves using the equation

(1) $$Y=Y_{\text{min}}+\frac{Y_{\text{max}}-Y_{\text{min}}}{10^{n*\left(\text{LogI}C_{50}-X\right)}+1}$$

where *Y*_min_ was the lower asymptote, *Y*_max_ was the upper asymptote, *n* was the Hill slope, and *IC*_50_ was the *x* value at the halfway point between *Y*_min_ and *Y*_max_ which represented the concentration of UNG2 that displaced half of the peptide from PCNA or RPA. During curve fitting, *Y*_min_ was constrained to its theoretical value of 0.0471 (for Pogo-Ligase peptide competition) or 0.0685 (for SMARCAL peptide competition); these were the mean anisotropy values of the free peptides in solution (Figure S1). The *IC*_50_ value from the competition assay using PCNA, Pogo-Ligase peptide, and UNG2(NR/DG) was used to a calculate a *K*_d_ for the PCNA—UNG2(NR/DG) interaction (termed *K*_i_ below) using the equation

(2) $$K_{i}=\frac{IC_{50}}{\left(\frac{L_{50}}{K_{d}}\right)+\left(\frac{p_{0}}{K_{d}}\right)+1}$$

where *L*_50_ was the concentration of free Pogo-Ligase peptide at 50 % inhibition, *P*_0_ was the concentration of free PCNA monomer at 0 % inhibition, and *K*_d_ was the dissociation constant of the PCNA—Pogo-Ligase complex [26,30]. The *IC*_50_ value from the competition assay using RPA32-WH, SMARCAL peptide, and UNG2(FF/AA) was used to a calculate a *K*_d_ for the RPA32-WH—UNG2(FF/AA) interaction using the same method. When used in competition assays, the fluorescein-labeled peptides were used at concentration of 50 nM, PCNA was used at a concentration of 1 μM, and RPA32-WH domain was used at a concentration of 20 μM.

### 2.4. Generation of HT29 UNG-KO cells

A mammalian expression plasmid called PX459 that constitutively expresses Cas9 and confers cellular resistance to puromycin was obtained from addgene (#48139) [31]. A guide RNA (gRNA) sequence targeting the UNG gene was inserted into the PX459 plasmid by restriction enzyme cloning using *Bbs*I sites [31]. The target sequence of the gRNA was 5′-GCGGCCCGCAACGTGCCCGT-3′ which targets both human isoforms of UNG (mitochondrial UNG1 and nuclear UNG2) [32]. HT29 cells were seeded at a density of 400,000 cells per well in 6-well plates in 2 ml of transfectagro media (Corning #40–300-CV). The next day, the cells were transfected with the PX459 plasmid by adding to each well 300 μl of transfectagro containing 6 μl of lipofectamine 2000 (Invitrogen #11668027) and 1.5 μg of plasmid DNA. The next day, the media was replaced with full DMEM for 24 hr to allow for recovery from transfection and expression of the puromycin resistance gene, then full DMEM containing 1 μg/ml of puromycin was added for 9 days to select for cells that obtained the plasmid DNA. The low chemical transfection efficiency of HT29 cells (less than 2 %) coupled with the strong puromycin selection left less than 10 cells per well, some of which began to form single cell colonies. After washing with PBS, the individual cell colonies were manually aspirated under a microscope using a 10 μl pipet and transferred to separate wells of a 96-well plate in full DMEM with 1 μg/ml of puromycin. 5 days later, the media was replaced with full DMEM containing 0.5 μg/ml of puromycin. 7 days later, cell colonies were proliferating in twenty wells of the 96-well plate. These were trypsinized and transferred back into 6-well plates in full DMEM to propagate the colonies, then cell lysates were prepared to analyze for the presence of UNG2 by western blot and enzyme activity assays.

### 2.5. Generation of stable HT29 UNG-KO cell lines that re-express UNG2 variants

The wild-type UNG2 gene was amplified by PCR with restriction enzyme sites on the N-terminus (*Bsi*WI) or the C-terminus (*Not*I) then inserted into an empty mammalian expression plasmid called pIR-ESneo3 (Takara #631621), which confers G418 antibiotic resistance to cells. We then used standard QuikChange/site directed mutagenesis to introduce six synonymous point mutations into the UNG2 gene at the location of the gRNA target sequence to ensure that the re-introduced gene was not targeted by residual Cas9 in the cells, thus yielding the final UNG2 re-expression plasmid. We then introduced point mutations into the gene to yield UNG2(FF/AA) and UNG2(NR/DG) re-expression plasmids.

For transfection, UNG-KO cells were first plated and grown for 24 hr in transfectagro media. Subsequently, the cells were trypsinized, pelleted, washed with PBS, and counted before resuspension in Buffer R, which is part of the Invitrogen Neon Electroporation System. We followed the manufacturer’s instructions for electroporating adherent cells with 2 μg of plasmid DNA using a 10 μl Neon Tip in 6-well plate format. 24 h after electroporation, the media was replaced with full DMEM containing 1 mg/ml of G418 to select for cells that obtained the plasmid. Individual cell colonies began to appear 7–10 days after the start of G418 selection. These cells were trypsinized and reseeded in 96-well plates at a density of 1 cell per well, the cell colonies were propagated in full DMEM, then cell lysates were prepared to analyze for the presence of UNG2 by western blot and enzyme activity assays.

### 2.6. Western blot

Cells were washed in their plate with PBS, then ice-cold RIPA buffer supplemented with protease inhibitors was added to the cells and incubated for 20 min on ice. The cells were scraped, and the supernatant was collected after centrifugation at 15,000× g for 15 min. The protein concentration was determined using a detergent-compatible Bradford protein assay (Thermo Fisher #23246). 5–10 μg of protein was analyzed by SDS-PAGE on Bio-Rad precast 4–15 % gels. Proteins were transferred to PVDF. This was blocked with 5 % BSA in TBS-T at room temperature then incubated with the primary antibody diluted 1:1000 in TBS-T overnight at 4 °C. A horseradish peroxidase-conjugated secondary antibody was then used at a 1:3000 dilution in TBS-T. Blots were developed by chemiluminescence (Thermo Fisher #34579) and imaged using an Azure Biosystems C400 imager.

### 2.7. Uracil excision assays

Uracil excision assays were performed using a synthetic, 87 bp dsDNA substrate that contained a single U/A bp near the middle of the duplex and a fluorescein end-label on the uracilated strand [17]. ~1.5 μg of protein from cell lysates prepared in RIPA buffer was diluted in a 15 μl final volume with 7.5 μM of the dsDNA substrate using 10 mM Tris-Cl (pH 8.0), 100 mM NaCl, 0.1 mM EDTA, and 1 mM DTT. Uracil excision reactions proceeded for 10 min at 37 °C before being quenched with NaOH (200 mM final concentration) and heat (95 °C for 10 min). Quenched reactions were diluted three-fold with formamide containing 5 mM EDTA, then the DNA fragments were separated by denaturing urea-TBE PAGE as described elsewhere [16–18,33,34]. Fluorescein-labeled oligonucleotides that represented the uracilated substrate and the excision product were visualized in the polyacrylamide gels with an Azure Biosystems C400 imager.

### 2.8. Cell viability assays

For assays using the yellow tetrazolium dye MTT, cells were initially seeded at a density of 10,000 cells per well in a 96-well plate in full DMEM. 24 h later, cells were treated with the indicated ligands dissolved in fresh media for 72 h; DMSO was used to prepare the ligands and its final concentration in the media was always 1 %. After the 72 h treatment, MTT was added followed by a formazen crystal-dissolving solution 3–4 hr later, according to the instructions in the Cayman Chemical assay kit (catalog #10009365). Absorbance in the wells was measured with a BioTek Synergy H1 plate reader at 570 nm. Cell viability was determined by dividing the absorbance values from the wells receiving drug treatment by the absorbance from control wells receiving DMSO treatment only, with a maximum value of 1, then this value was multiplied by 100 %. The percent cell viability was plotted as *y* values against the log_10_ of the drug dose as *x* values, and a curve was fit to the data using Eq. 1 with *Y*_max_ constrained to 100. The *IC*_50_ values that were determined from the fit curves were relative *IC*_50_ values, representing the halfway point between the two asymptotes, as opposed to absolute *IC*_50_ values, which was the dose where 50 % of the cells were viable. The relative *IC*_50_ values were problematic for interpreting drug potency, so we used the fit curves to calculate by interpolation the absolute *IC*_50_ values instead which we reported on the figure panels.

For trypan blue dye exclusion assays, cells were seeded at a density of 10,000 cells per well in a 24-well plate. 24 h later, cells were treated with the indicated ligands dissolved in fresh media for 72 h; the final DMSO concentration in the media was 1 %. After the treatment, cells were washed with PBS, trypsinized, resuspended in PBS, then mixed 1:1 with 0.4 % trypan blue solution. The cells were counted on a slide according to the manufacturer’s instructions using an Invitrogen Countess 3 Automated Cell Counter. Cell viability was determined as the percent of live cells divided by the total number of counted cells.

### 2.9. Cloning, expression, and purification of recombinant His-SUMO-UdgX

A plasmid containing the *Mycobacterium smegmatis* UdgX gene was obtained from addgene (catalog #163543) [35]. The UdgX gene was amplified by PCR with *Bsi*WI and *Not*I restriction enzyme sites flanking the N-terminus and C-terminus of the gene, respectively, and the amplicon was purified by agarose gel electrophoresis. Separately, we used a pET21a plasmid from our lab that encoded the protein 8xHis-SUMO-UNG2 [26]. We used standard QuikChange/site directed mutagenesis to insert a *Bsi*WI site into the 8xHis-SUMO-UNG2 plasmid immediately downstream of the SUMO domain, and the pET21a plasmid already contained a *Not*I site downstream of the UNG2 gene. Standard restriction enzyme cloning replaced the UNG2 gene with UdgX to yield 8xHis-SUMO-UdgX with a residual *Bsi*WI site between the SUMO domain and UdgX, which we left in place to encode presumably inert arginine and threonine residues. All cloning was confirmed by Sanger sequencing.

The pET21a plasmid encoding 8xHis-SUMO-UdgX was transformed into BL21(DE3)pLysS cells, plated on LB agar plates containing 50 mg/L of ampicillin, then colonies were picked for overnight starter cultures at 37 °C. The starter cultures were used to inoculate flasks containing 1 L of LB media and 50 mg/L ampicillin; our standard conditions for bacterial growth and protein expression have been reported previously [26]. When the optical density reached ~0.6, IPTG was added to a final concentration of 0.25 mM along with 0.01 % FeCl_3_, and the flasks continued to shake at 21 °C for 16 h.

After growth, the bacteria were pelleted and resuspended in ice-cold lysis buffer containing 20 mM Tris-Cl (pH 7.5), 300 mM NaCl, 20 mM imidazole, and 1 mM TCEP along with protease inhibitors. Cells were lysed with a Microfluidics LM10 Microfluidizer, 8xHis-SUMO-UdgX was captured on a Ni^2+^ column (Cytiva catalog #17531802), then the protein was eluted with a step gradient of imidazole up to 500 mM dissolved in lysis buffer [26]. Fractions containing 8xHis-SUMO-UdgX were pooled, diluted five-fold with Buffer A (20 mM Tris-Cl (pH 7.5) and 1 mM DTT), and the protein was further purified using anion exchange chromatography [36]. After binding to the Q column (Bio-Rad catalog #7800003), the protein was eluted with a linear gradient of NaCl dissolved in Buffer A. Fractions containing 8xHis-SUMO-UdgX were analyzed by SDS-PAGE, pooled, and stored as aliquots at −80 °C for future use. The concentration of the protein was estimated by its absorbance at 280 nm where 1 absorbance unit was equal to 1 mg/ml.

### 2.10. Measurement of genomic uracil levels using UdgX dot blot

Cells were seeded in 6-well plates at a density of 500,000 cells per well in full DMEM. 24 hr later, fresh media was added containing 100 nM FdU, 100 nM pemetrexed, or 1 % DMSO, which controlled for the ligands’ solvent. After 48 hr of treatment, the cells were trypsinized and pelleted to extract genomic DNA using the New England Biolabs Genomic DNA Purification Kit (catalog #T3010S). 0.6 μg of genomic DNA from each condition was diluted with 1.4 μg of salmon sperm carrier DNA in water. A positively charged nylon membrane from Cytiva (Amersham Hybond-N+) was prewet with SSC buffer (150 mM NaCl and 15 mM sodium citrate, pH 7.0), then the membrane was inserted into a Schleicher & Schuell Minifold I vacuum system with a 96-well format. The spots on the membrane were pre-washed in the vacuum system with 10 mM Tris-Cl (pH 8.0), 100 mM NaCl, and 0.1 mM EDTA, then the genomic DNA samples were spotted onto the membrane using the vacuum. After briefly washing the membrane with SSC buffer, the membrane was baked for 2 hr at 60°C in an incubator. The membrane was blocked for 1 hr at room temperature with a 10 ml solution containing modified TBS-T (25 mM Tris-Cl (pH 7.5), 2.7 mM KCl, 137 mM NaCl, 1 mM EDTA, and 0.05 % Tween-20) with 5 % BSA (w/v) and 100 μg of salmon sperm DNA [37]. Then, the membrane was incubated overnight at 4 °C with a solution of modified TBS-T containing 3 μg/ml of His-SUMO-UdgX and 5 % BSA. The next day, the membrane was washed with TBS-T five times at room temperature for 10 min each followed by incubation with a 6xHis tag antibody at a 1:1000 dilution in TBS-T for 1.5 h at room temperature. After removing the primary antibody and washing with TBS-T, the blot was incubated with a secondary antibody at a 1:3000 dilution for 1 h at room temperature. The blot was developed by chemilumenscence and imaged using an Azure Biosystems C400 imager. Spot intensity was then quantified using Fiji/ImageJ software [38]. To account for variability in spot intensity between blots, we normalized each drug-treated spot to the corresponding control spot from the same cell line and blot, yielding a fold change relative to DMSO-treated cells. We did not test whether the signal was in a linear range as a function of uracil base concentration or if the fold change was semi-quantitative. For statistical analysis, we used a one-sample *t*-test to determine whether the fold change differed significantly from 1, which represented no change in intensity compared to control.

### 2.11. Comet assay

Cells were seeded at a density of 500,000 cells per well in 6-well plates. 24 hr later, the cells were treated with fresh media containing FdU, pemetrexed, or DMSO for 48 hr; the final DMSO concentration in the media was 1 %. Subsequently, the cells were trypsinized, embedded in agarose on CometSlides, lysed, and subjected to alkaline electrophoresis as described previously [39]. We used a Trevigen CometAssay ES II electrophoresis apparatus and buffer solutions supplied by the same manufacturer, and we stained the DNA with GelGreen prior to imaging with a Zeiss LSM 800 confocal microscope. Images were analyzed using the OpenComet plugin for Fiji/ImageJ [38–40], and the data in the output files were manually verified by inspecting the images. The Olive Tail Moment was calculated as the percent of DNA in the tail multiplied by the distance between the centers of mass of the comet head and tail [41]. For the FdU treatments, the non-normal datasets were analyzed with a Kruskal-Wallis test, which is a nonparametric analog of a one-way ANOVA, followed by Dunnett’s multiple comparisons test comparing the control (DMSO-treated) to the FdU-treated data within each cell line. For the pemetrexed treatments, we analyzed the data with a Kruskal-Wallis test followed by Dunnett’s multiple comparisons test comparing the damage in the HT29 cells (pemetrexed-treated) to the damage in the other cell lines treated with the same dose. All statistical analyses in this work were performed with GraphPad Prism v10.

### 2.12. Immunofluorescence using confocal microscopy

Cell lines were seeded on coverslips in a 6-well plate at a density of 100,000 cells per well. The cells were synchronized by serum starvation in DMEM for 48 hr. Upon their release into full DMEM, the cells were treated with FdU, pemetrexed, or 1 % DMSO as a control until the specified time point. The cells were then washed with PBS and incubated in 1 ml of fresh 4 % paraformaldehyde at room temperature for 10–12 min with rotation. Crosslinking was quenched by replacing the paraformaldehyde with 30 mM glycine in PBS (pH 7.5) for 5 min, followed by a wash with PBS. Cells were permeabilized using PBS with 0.25 % Triton X-100 for 15 min at room temperature, again with rotation, followed by a wash with PBS and blocking in 1 ml of 2 % BSA in PBS for 1 hr at room temperature. After washing twice with PBS for 5 min each, primary antibodies were applied in PBS overnight using 1:500 dilutions. After washing three times with PBS, the secondary antibodies were added at 1:1000 dilutions for 1 hr at room temperature. After washing three times with PBS, the coverslips were mounted on glass slides with VECTASHIELD antifade mounting medium with DAPI (Vector Laboratories). Microscopy images were collected with a Zeiss LSM 800 laser scanning confocal microscope equipped with the Airyscan super-resolution detector system and a 63x oil immersion objective. For multichannel imaging in standard Airyscan SR mode, the following excitation lasers and fluorophores were used: 488 nm for Alexa Fluor 488, 561 nm for Alexa Fluor 594, and 640 nm for Alexa Fluor 647. The detector wavelengths were 471–575 nm for Alexa Fluor 488 (emission wavelength of 517 nm), 559–631 nm for Alexa Fluor 594 (emission wavelength of 618 nm), and 584–700 nm for Alexa Fluor 647 (emission wavelength of 668 nm). Imaging was performed using 2 by 2 tiled fields to increase the number of cells in the focal plane. Parameters such as detector gain, laser power, scan speed, and pinhole size were kept constant across all conditions. Post-acquisition, Airyscan images were processed using the built-in method in Zen Blue software to improve resolution and signal-to-noise ratio. All images were exported with individual channels in TIFF format. Brightness and contrast adjustments for comparison images were applied uniformly across control and experimental groups. In ImageJ, each channel was opened, adjusted if needed, and merged into RGB TIFF files. Images were analyzed with Fiji/ImageJ for detecting replication foci [38]. Cells with more than 10 punctate foci were counted, and this number was then compared to the total cell count to calculate the percentage of cells exhibiting foci.

## 3. Results

### 3.1. Design and generation of stable cell lines to study UNG2 protein-protein interactions

**Fig. 2.**
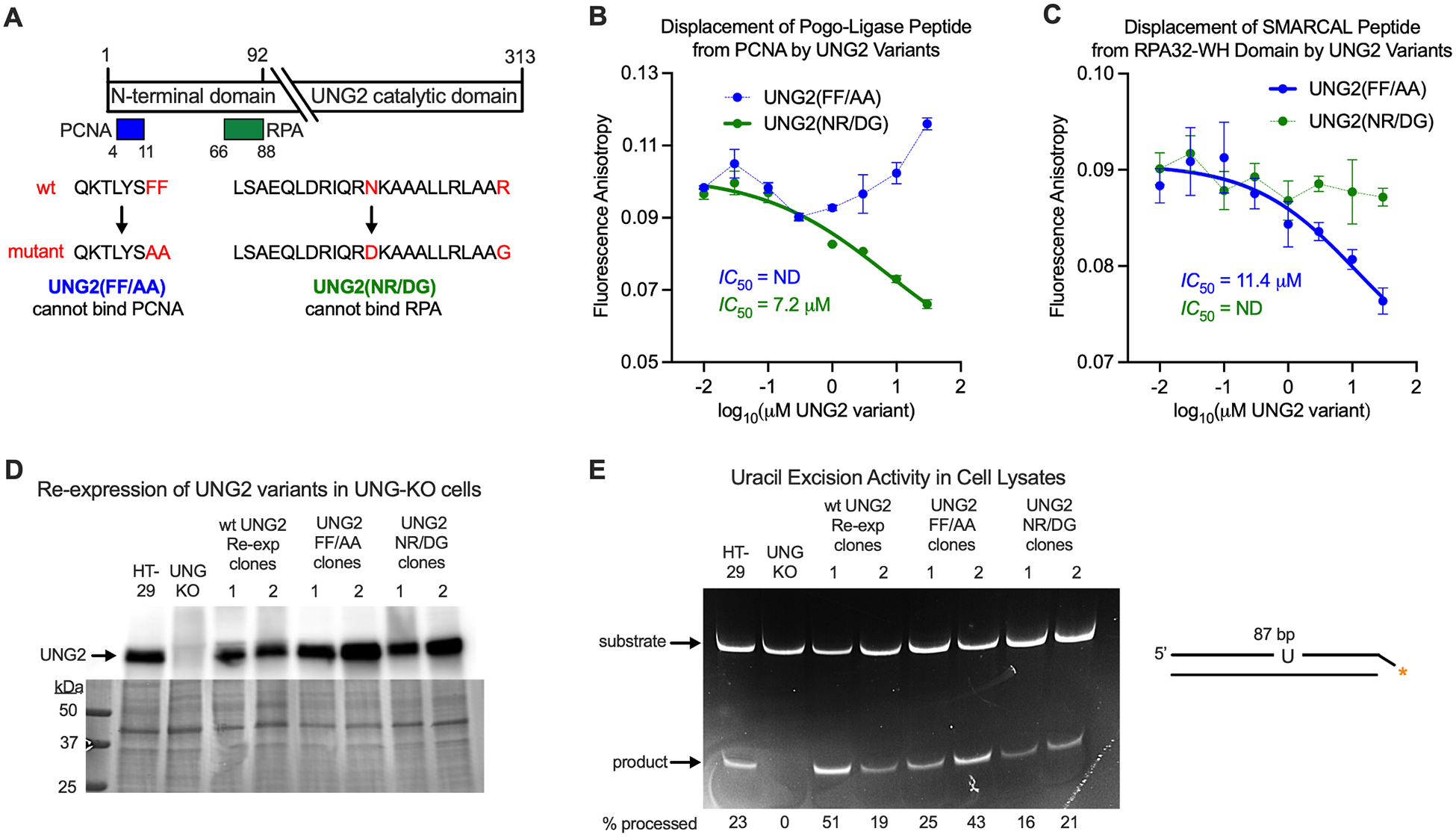
Design and generation of stable cell lines to study UNG2 protein-protein interactions. (A) Scheme of UNG2 with the location of PCNA and RPA binding sites indicated. The mutant variants UNG2(FF/AA) and UNG2(NR/DG) have the sequences shown. (B) Recombinant UNG2(FF/AA) could not interact with PCNA and displace Pogo-Ligase, which is a synthetic peptide that binds to the same site on PCNA as wild-type UNG2 [26]. UNG2(NR/DG) interacted normally with PCNA and competitively displaced the peptide with an *IC*_50_ = 7.2 μM (ND, not determined). (C) Recombinant UNG2(FF/AA) interacted normally with the RPA32-WH domain and competitively displaced a synthetic peptide called SMARCAL from the same site with an *IC*_50_ = 11.4 μM [26]. UNG2(NR/DG) could not interact with RPA32-WH and displace the bound peptide (ND, not determined). (D) Here we show a UNG2 western blot of various cell lysates with the protein indicated by the arrow, and beneath it, we show the Coomassie-stained membrane from the western as a loading control. For reference, UNG2 protein is 35 kDa. (E) An 87 bp, uracil-containing dsDNA substrate was added to cell lysates to measure uracil excision activity. Subsequently, the reactions were quenched with heat and NaOH, which also cleaved the abasic sites generated by enzymatic uracil excision. Denaturing urea-TBE PAGE was then used to separate the longer substrate oligonucleotide from the shorter product, which were detectable in the gel because of a fluorescein end-label on the oligonucleotides. The percent of substrate processed for each condition in this representative assay is shown beneath the gel.

To study how protein interactions affect UNG2 activity in cells, including during treatment with various TS inhibitors, we aimed to generate stable cell lines that expressed wild-type UNG2, no UNG2, or UNG2 variants that were incapable of binding to PCNA or RPA. UNG2 interactions with PCNA and RPA are thought to be functionally independent, and mutation of one binding interface should not impact the other [19,26]. To demonstrate this, we designed the variant UNG2 (FF/AA) with two mutations on the N-terminus of the protein to disrupt its interaction with PCNA, which occurs through a canonical “PIP-box” motif on UNG2 (Fig. 2A) [26,42,43]. Alternatively, UNG2(NR/DG) has two mutations that were designed to disrupt the binding surface between UNG2 and the winged-helix domain of RPA32 (RPA32-WH), which is a well-characterized protein binding domain on RPA (Fig. 2A) [26,44,45]. To validate the mutations *in vitro*, we expressed and purified recombinant UNG2(FF/AA) and UNG2(NR/DG) for binding assays (Figure S1). In one experiment, we complexed purified PCNA with a peptide called Pogo-Ligase that also contains a PIP-box motif and binds to the same site on PCNA as wild-type UNG2 (Figure S1) [26,46]. UNG2 (FF/AA) was unable to bind PCNA and displace Pogo-Ligase from the site, whereas UNG2(NR/DG) displaced the peptide from PCNA with an *IC*_50_ of 7.2 μM (Fig. 2B). We used the *IC*_50_ value and Eq. 2 to calculate a *K*_d_ value of 0.4 μM for the interaction of UNG2(NR/DG) with PCNA (see Section 2.3 in the Materials and Methods). In the same assay, we previously showed that wild-type UNG2 and PCNA interacted in the same range with a *K*_d_ of 1 μM [26], further validating that UNG2(NR/DG) retained the ability to bind PCNA. In a second experiment, we complexed recombinant RPA32-WH domain with a peptide called SMARCAL that binds to the same site on RPA as wild-type UNG2 (Figure S1). UNG2 (FF/AA) displaced SMARCAL peptide from the RPA32-WH domain with an *IC*_50_ of 11.4 μM, while UNG2(NR/DG) was unable to bind competitively with the peptide (Fig. 2C); we used the *IC*_50_ and Eq. 2 to calculate a *K*_d_ value of 4.6 μM for the interaction of UNG2(FF/AA) with the RPA32-WH domain. As a close comparison, we previously determined a *K*_d_ value of 3 μM for the interaction of wild-type UNG2 with full-length RPA using the same assay [26]. These experiments validated that UNG2 (FF/AA) and UNG2(NR/DG) were selectively incapable of binding to PCNA and RPA, respectively.

To study the activity of UNG2 and its variants in cells, we chose HT29 colorectal cancer cells as our model because they were reported to be heavily reliant on UNG2 during treatment with TS inhibitors, and in the absence of UNG2 activity, HT29 cells become hypersensitive to uracil lesions [5,13]. First, we used CRISPR/Cas9 to knockout the UNG gene in parental HT29 cells, which was confirmed by western blot (Fig. 2D and Figure S2). Then we stably re-expressed the variants UNG2(FF/AA) or UNG2(NR/DG) in the UNG-KO cells by transfecting expression constructs and selecting with antibiotics to promote stable genomic integration through recombination (Fig. 2D). We also re-expressed wild-type UNG2 in the UNG-KO cells to serve as a control because the re-introduced proteins were expressed under an exogenous CMV promoter (Fig. 2D), whereas expression of native UNG2 varies throughout the cell cycle [25]. As shown later in the Results Section 3.4, all of the re-expressed UNG2 variants localized to the nucleus similar to wild-type UNG2 from parental HT29 cells.

In crude assays using whole cell lysates, we detected negligible uracil excision activity in HT29 UNG-KO cells in contrast to the parental HT29 cells, where uracil was robustly removed from a synthetic dsDNA substrate (Fig. 2E). Likewise, we detected robust uracil excision activity in lysates from multiple clones of UNG-KO cell lines re-expressing UNG2 (FF/AA), UNG2(NR/DG), or wild-type UNG2 (Fig. 2E). We concluded that UNG2 and its variants that were incapable of binding PCNA or RPA were stably re-expressed in their cell lines, folded correctly, and enzymatically functional in solution conditions. Lastly, it is worth noting that extended exposure of synthetic dsDNA to higher concentrations of cell lysate did identify a low level of uracil excision in UNG-KO cells that was ~99 % slower than the parental HT29 cells (Figure S2). The residual activity was attributed to less efficient enzymes such as SMUG1 or TDG which are capable of excising uracil from U/A base pairs [3,4].

### 3.2. Role of UNG2 in determining sensitivity of cells to TS inhibitors

**Fig. 3.**
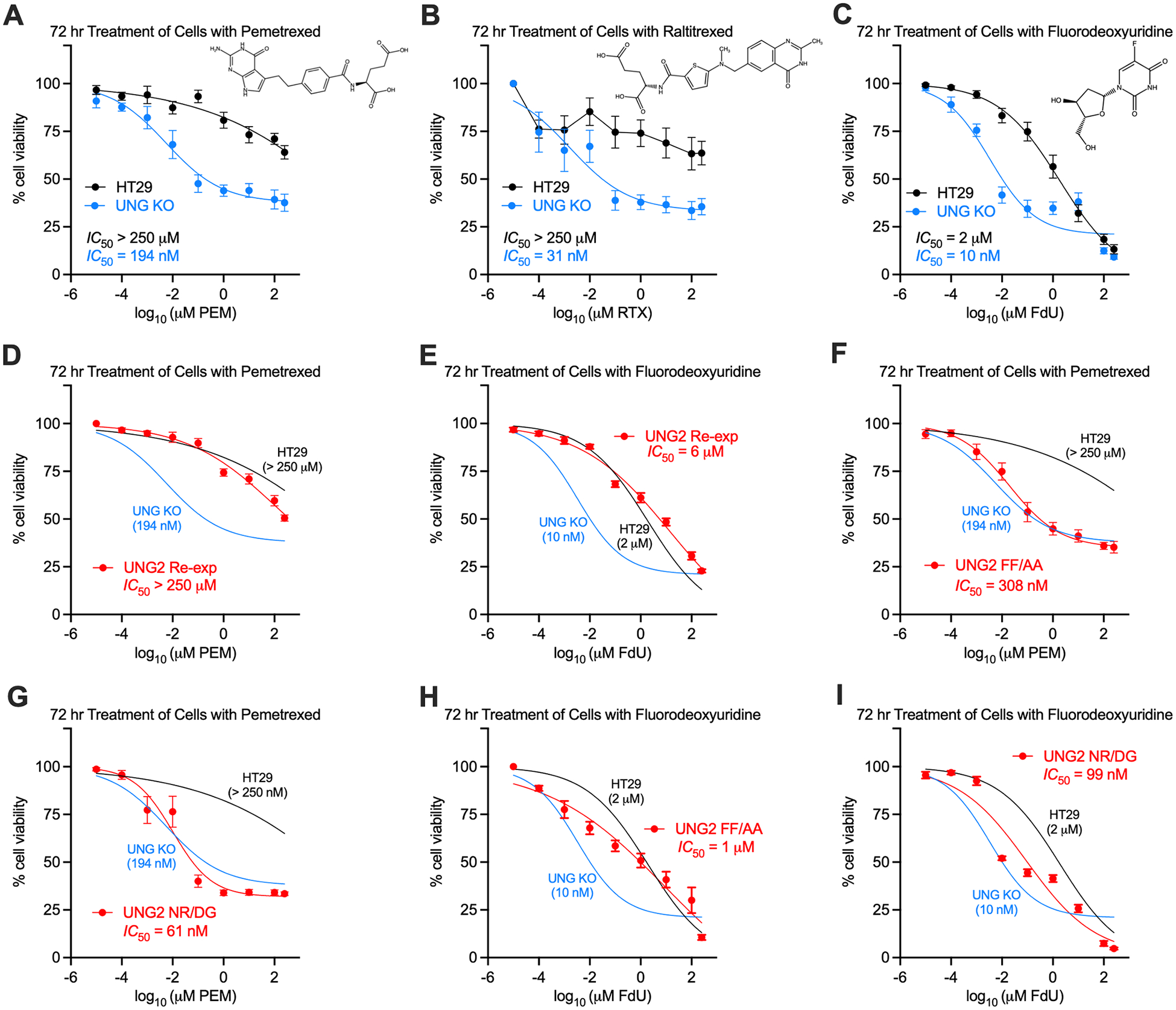
Sensitivity to TS inhibitors was determined by UNG2 and its ability to interact with PCNA and RPA. (A) Sensitivity of HT29 and HT29 UNG-KO cells to pemetrexed as determined with an MTT cell viability assay. In all panels, the *IC*_50_ represents the dose that inhibited cell viability by 50 % as determined by the fit curve. In all panels, points represent the mean ± standard error from at least six independent experiments. (B) Sensitivity of HT29 and UNG-KO cells to raltitrexed using the methods described above for panel A. (C) Sensitivity of HT29 and UNG-KO cells to FdU. (D) The hypersensitivity of UNG-KO cells to pemetrexed was reversed by re-expression of wild-type UNG2. (E) The hypersensitivity of UNG-KO cells to FdU was reversed by re-expression of wild-type UNG2. (F) Re-expression of UNG2(FF/AA), which cannot bind PCNA, does not reverse the hypersensitivity of UNG-KO cells to pemetrexed. (G) Re-expression of UNG2(NR/DG), which cannot bind RPA, does not reverse the hypersensitivity of UNG-KO cells to pemetrexed. (H) Re-expression of UNG2(FF/AA) in UNG-KO cells only partially reversed their hypersensitivity to FdU. (I) Re-expression of UNG2(NR/DG) in UNG-KO cells partially reversed their hypersensitivity to FdU.

We treated our cell lines with TS inhibitors to understand how UNG2 and its protein interactions influence the ability of cells to combat toxicity caused by genomic uracilation. HT29 UNG-KO cells were at least 1000-fold more sensitive to pemetrexed and raltitrexed than the parental HT29 cells (Fig. 3A and B). Likewise, the potency of FdU increased 200-fold in the UNG-KO cell line (Fig. 3C). Consistent with literature reports [5,12], the UNG-KO cell line was not hypersensitive to treatment with 5-fluorouracil alone, which is a cancer therapy with a different mechanism of action from FdU (Figure S3). These measurements of cell viability were made indirectly using an MTT assay that reported on the metabolic activity of treated cells. We confirmed that the UNG-KO cells were hypersensitive to FdU and pemetrexed using a trypan blue dye exclusion assay to measure cell viability (live vs. dead cells) (Figure S4).

Importantly, re-expression of wild-type UNG2 in UNG-KO cells produced cell lines that were indistinguishable from the parental HT29 cells. We focused our investigation on pemetrexed and FdU because of their different mechanisms of action (Fig. 1A) and to streamline the drug/cell line combinations examined with other approaches. Wild-type UNG2 rescued the UNG-KO cells from their hypersensitive response to pemetrexed and FdU (Fig. 3D, E, and S4). In contrast, the cell lines expressing UNG2(FF/AA) or UNG2(NR/DG), which were incapable of binding to PCNA or RPA, remained strongly hypersensitive to pemetrexed (Fig. 3F, G and S4). We also determined that UNG2(FF/AA) and UNG2(NR/DG) were not able to fully rescue UNG-KO cells from their hypersensitive response to FdU (Fig. 3H, I, and S4). We concluded that disrupting the protein-protein interactions of UNG2 made the protein deficient in its ability to protect cells from the cytotoxic effects of TS inhibitors.

### 3.3. DNA repair is deficient when UNG2 cannot interact with PCNA and RPA

**Fig. 4.**
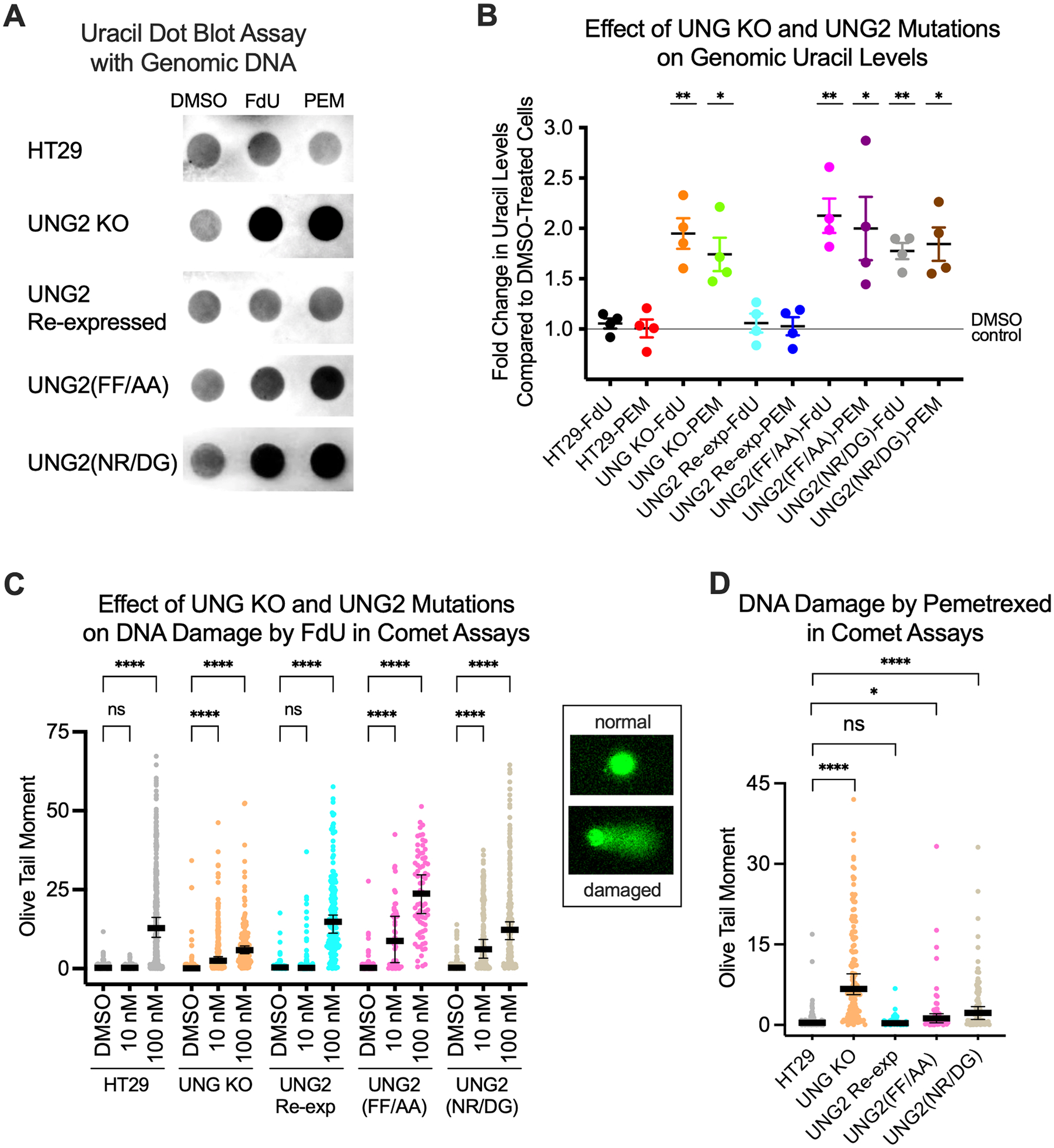
Increased uracil levels and DNA damage in cells treated with TS inhibitors when UNG2 does not interact with PCNA or RPA. (A) Dot blots showing increased uracil levels in genomic DNA when cells were treated with 100 nM FdU or 100 nM pemetrexed (PEM) for 48 hr. Uracil bases in genomic DNA were covalently attached to UdgX, which was detected with an antibody and chemiluminescence. (B) Quantification of uracil levels in cell lines from dot blots as in panel A. Intensities from FdU- or pemetrexed-treated spots were normalized to an adjacent control spot intensity from the same cell line and blot. This yielded a fold change relative to DMSO-treated control cells which have a normalized value of 1. Asterisks indicate that uracil levels in the treatment groups were significantly elevated compared to DMSO-treated cells for the same cell line (analyzed with a one-tailed *t*-test; *, p < 0.05; **, p < 0.01). We show mean ± standard error from four biological replicates. (C) Measurement of DNA fragmentation by comet assay for different cell lines and FdU concentrations. Normal and damaged DNA was detected by separating genomic DNA from individual cells by electrophoresis and staining with GelGreen; representative images of normal and damaged cellular DNA are shown beside the graph. DNA damage was quantified by calculating the Olive tail moment (see Section 2.11 in the Materials and Methods). Each point in the graph represents a single cell (each group had at least 46 cells across two independent experiments). The black bars indicate the median and the 95 % confidence interval. The non-normal data was analyzed with a Kruskal-Wallis test (p value < 0.0001) followed by Dunnett’s multiple comparisons tests for the specific pairs indicated by the brackets (****, p < 0.0001). (D) Measurement of DNA fragmentation by comet assay for cell lines treated with 100 nM pemetrexed. Each group had at least 45 cells across two independent experiments, and the black bars indicate the median and the 95 % confidence interval. The data was analyzed with a Kruskal-Wallis test (p value < 0.0001) followed by Dunnett’s multiple comparisons tests for the specific pairs indicated by the brackets (****, p < 0.0001; *, p < 0.05).

We measured DNA damage in cells after treatment with FdU or pemetrexed to confirm how UNG2 plays a protective role against DNA lesions with its protein binding partners. The treatment dose that we chose (100 nM) can become quite toxic to certain cell lines after 72 h (Fig. 3), so we chose a shorter time point where cell viability still exceeded 50 % (48 h). We developed a dot blot assay to quantify levels of uracil bases in genomic DNA following treatment of cells with TS inhibitors. In this method, cellular DNA was purified and spotted on a membrane, then uracil bases in the genomic DNA were reacted with the bacterial enzyme UdgX, which covalently attaches to uracil-containing DNA (Figure S5) [47]. UdgX was then detected on the blot, bound to genomic uracil, with an antibody directed towards its 8xHis tag. Compared to the parental HT29 cells, UNG-KO cells had significantly elevated levels of uracil bases in genomic DNA following treatment with FdU or pemetrexed (Fig. 4A and B). Re-expression of wild-type UNG2 prevented the accumulation of uracil bases during treatments (Figs. 4A and 4B). In contrast, cells expressing UNG2 variants that were incapable of binding to PCNA or RPA had elevated genomic uracil after TS inhibitor treatment (Figs. 4A and 4B).

We further examined DNA damage in cells after treatment with FdU or pemetrexed using a comet assay, which detects DNA strand breaks and other lesions such as abasic sites that are converted into breaks under alkaline conditions. In this assay, fragmented DNA produces a “comet tail” during whole-cell electrophoresis (Fig. 4C, *boxed images*) [48]. Treatment of parental HT29 cells with 100 nM FdU produced fragmented DNA (Fig. 4C, *scatter plot*). This damage could arise from unwound DNA or abasic sites that result from successful excision of uracil and would not be detected with the UdgX assay. Compared to the parental HT29 cells, the cells expressing UNG2 variants that were incapable of binding to PCNA or RPA were much more sensitive to the DNA-damaging effects of FdU, as were the UNG-KO cells, which had detectable DNA strand breaks at 10 nM FdU (Fig. 4C, *scatter plot*). The UNG-KO cells that were re-expressing wild-type UNG2 behaved similar to the parental HT29 cells and required 100 nM FdU for significant DNA damage (Fig. 4C).

The parental HT29 cells and the cell line re-expressing wild-type UNG2 were not very susceptible to DNA fragmentation after treatment with 100 nM pemetrexed (Fig. 4D), which was in contrast to the DNA damage that we observed after treatment with 100 nM FdU (Fig. 4C). This was consistent with FdU being the more potent ligand in cytotoxicity experiments (Figs. 3A and 3C). Nonetheless, 100 nM pemetrexed was sufficient to induce DNA damage in UNG-KO cells and cells expressing UNG2(FF/AA) or UNG2(NR/DG) (Fig. 4D). These experiments demonstrated that UNG2 required interactions with both PCNA and RPA to efficiently combat the DNA-damaging effects of TS inhibitors.

### 3.4. Efficient replication fork localization for UNG2 during DNA repair requires both PCNA and RPA interactions

We examined how PCNA and RPA affect the distribution of UNG2 in the nucleus and its ability to localize to the replication fork when DNA damage occurs. Reports indicate that PCNA and RPA are relatively diffuse in the nucleus of untreated cells, although some discrete nuclear foci containing PCNA may form during S-phase because of its tight association with the replisome [49–51]. In contrast, RPA is much more dynamic and associates transiently with replication forks in unperturbed cells [49–51]. Upon treatment with DNA damaging agents, both PCNA and RPA accumulate at stalled replication forks to produce discrete foci throughout the nucleus where DNA repair factors are recruited and stabilized [49–52]. An important question is whether PCNA and RPA redundantly recruit UNG2 to sites of DNA damage at stalled replication forks, or if the ability of UNG2 to form foci declines in efficiency when it can only bind to one partner.

**Fig. 5.**
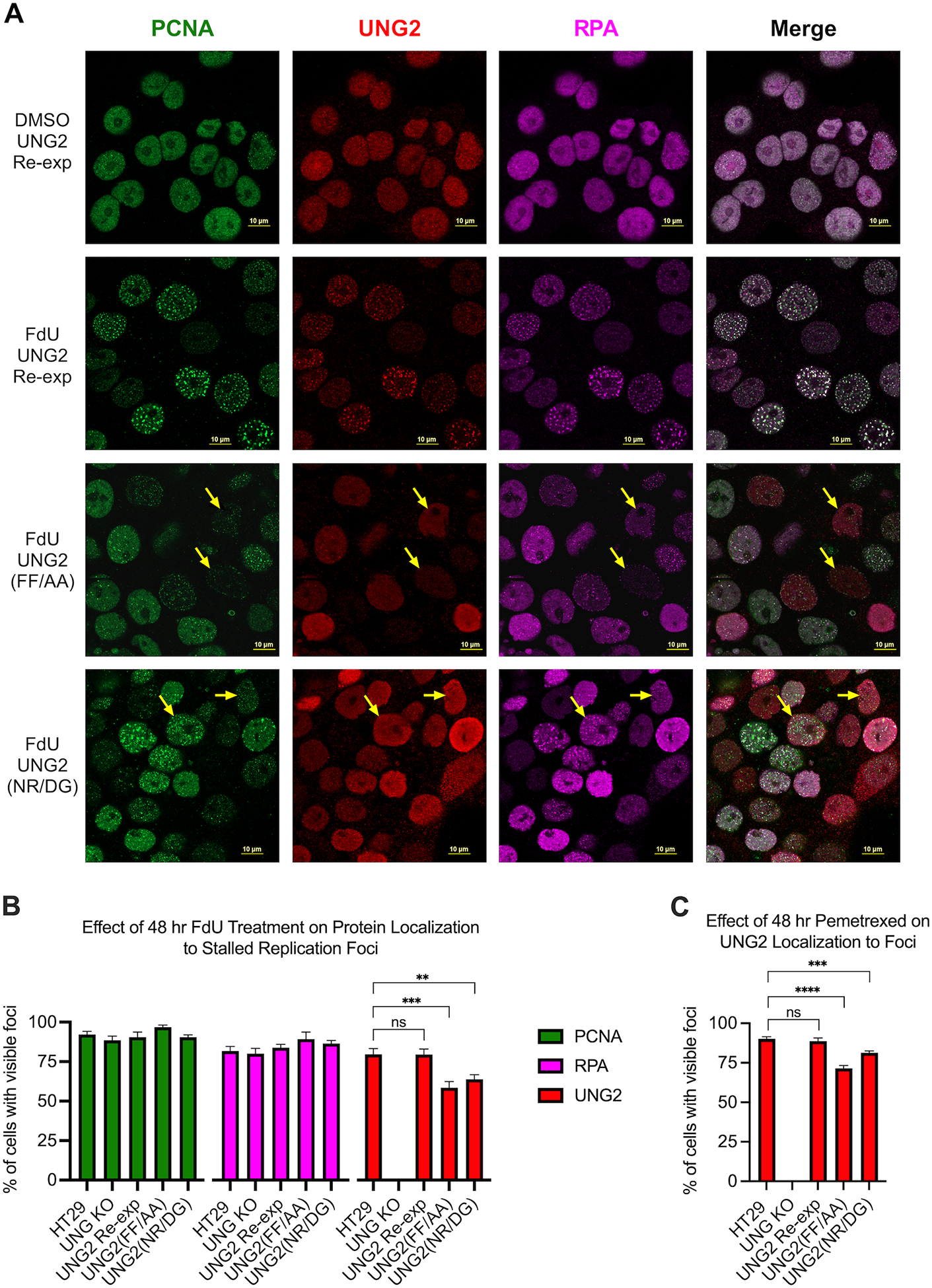
Ability of UNG2 and its variants to localize to the replication fork with PCNA and RPA during TS inhibitor treatments. (A) Confocal microscopy images of HT29 UNG-KO cell lines that were re-expressing wild-type UNG2 (Re-exp), UNG2(FF/AA), or UNG2(NR/DG). The cells were treated with DMSO vehicle or 100 nM FdU for 48 hr as indicated. During FdU treatment, RPA, PCNA, and wild-type UNG2 readily co-localized to discrete foci representing stalled replication forks. However, UNG2(FF/AA) and UNG2(NR/DG) sometimes failed to localize to the foci with PCNA and RPA (some examples of this are indicated with the yellow arrows). See Fig. S6 for additional images of the cell lines treated with DMSO or FdU. (B) The percent of cells showing discrete foci containing PCNA, RPA, or UNG2 was quantified from microscopy images after cells were treated with 100 nM FdU. The bar graphs represent the average percent of cells ± standard error in which each protein formed foci from seven to thirteen images from at least two independent experiments. Compared to UNG2 in the parental HT29 cells, the ability of UNG2(FF/AA) and UNG2(NR/DG) to localize to foci was significantly reduced as determined with a one-way ANOVA (p value = 0.0001) followed by Dunnett’s multiple comparisons tests for the specific pairs indicated by the brackets (**, p < 0.01; ***, p < 0.001). (C) The percent of cells showing discrete UNG2 foci was quantified from microscopy images after cells were treated with 100 nM pemetrexed (see Figs. S7 and S8 for representative images). The bar graphs represent the average percent of cells ± standard error in which each protein formed foci from twelve to fifteen images from at least two independent experiments. The ability of UNG2(FF/AA) and UNG2(NR/DG) to localize to foci was significantly reduced compared to UNG2 in the parental HT29 cells as determined with a one-way ANOVA (p value < 0.0001) followed by Dunnett’s multiple comparisons tests for the specific pairs indicated by the brackets (***, p < 0.001; ****, p < 0.0001).

We synchronized our HT29 cell lines by serum starvation and treated the cells with a DMSO vehicle or 100 nM FdU upon their release. In the absence of FdU treatment, UNG2 and RPA stained diffusely throughout the nucleus while only a few cells showed discrete PCNA foci, as expected (Fig. 5A, *top row*, and Figure S6). In contrast, PCNA and RPA localized to discrete nuclear foci within 48 hr in > 80 % of the cells that were treated with FdU, indicating widespread stalling of replication forks in every cell line (Fig. 5A, B, and S6). When wild-type UNG2 was being expressed in the cells, the enzyme also efficiently localized to the replication fork with PCNA and RPA at stalled replication forks (Fig. 5A, B, and S6). However, when the interaction of UNG2 with either PCNA or RPA was impaired, the enzyme was less likely to localize to the replication fork (Figs. 5A and 5B). It was clear that PCNA and RPA were able to localize to the stalled replications forks whether or not they interacted with UNG2 (Fig. 5A, B, and S6). In some cells expressing UNG2(FF/AA) or UNG2(NR/DG), the enzyme remained diffuse throughout the nucleus even while PCNA and RPA were together in the same foci (Fig. 5A). Other times, both UNG2(FF/AA) and UNG2(NR/DG) showed a normal capacity to localize to stalled replication forks (Figs. 5A and 5B). The heterogeneity suggested that UNG2 was not redundantly targeted by PCNA and RPA to damaged DNA at stalled replication forks, and that UNG2 relied on its binding partners in different contexts (see Section Discussion).

Finally, we synchronized our HT29 cell lines by serum starvation and treated the cells with 100 nM pemetrexed for 48 hr. The results were similar to our observations during FdU treatments (Fig. 5C, S7, and S8). PCNA, RPA, and wild-type UNG2 reliably localized to discrete nuclear foci after pemetrexed treatment (Fig. 5C, S7, and S8). UNG2(FF/AA) and UNG2(NR/DG) did not localize to the stalled replication forks as efficiently as the wild-type protein (Fig. 5C and S7). In many cells, the UNG2 variants remained diffuse in the nucleus even as PCNA and RPA formed discrete foci together (Figures S7). Thus, impairing the interactions of UNG2 with PCNA and RPA reduced its ability to localize to pemetrexed-induced DNA damage.

## 4. Discussion

In this work, we found that UNG2 played a key role in repairing genomic DNA in a colorectal cancer model after DNA damage was induced by TS inhibitors including pemetrexed and FdU. Further, UNG2 relied on its binding partners PCNA and RPA to optimize its enzymatic efficiency in cancer cells and counteract TS inhibitor toxicity. A key role for PCNA and RPA was to localize UNG2 to stalled replication forks in the nucleus, presumably to sites of DNA damage. Enzymology studies also show that PCNA and RPA enhance UNG2 activity when it arrives at forked DNA by targeting the enzyme to nearby uracil and 5-fluorouracil bases [16,17,19,53]; thus, the protein interactions serve a dual function for recruiting the enzyme then enhancing its activity where it is needed. We concluded that efficient activity of UNG2 in our model of proliferating cancer cells required binding to both PCNA and RPA.

Our data did not support a simplified model where PCNA recruits UNG2 to actively replicating foci, while RPA mediates the recruitment of UNG2 to stalled replication forks. Instead, our study supported the idea that the PCNA interaction can also recruit or retain UNG2 at specific sites of damage [43]. We speculate that the protein responsible for recruiting UNG2 depends on the context of the DNA damage. The lesion inducing fork arrest could conceivably be in the template strand, pre-replicative or post-replicative, as well as the newly synthesized strand (Fig. 1B). It is also possible that the type of lesion disrupting replication plays a role in how UNG2 is recruited (e.g., uracil bases, 5-fluorouracil bases, or abasic sites). These lesions can persist in the genome if not immediately corrected and become problematic when encountered during the next round of replication [13]. The recruitment of UNG2 to a stalled replication fork by PCNA or RPA is regulated, including by post-translational modifications [26,54,55], and is not part of a generalizable DNA damage response that occurs for all base lesions.

Our study has implications for how cancers are treated with thymidylate inhibitors including pemetrexed, FdU, and raltitrexed, which are widely used clinically in humans. First, depletion of UNG2 activity in cells can significantly increase the potency and efficacy of TS inhibitors, which supports the development of UNG2 inhibitors as therapeutic sensitizers that increase the toxicity associated with uracil lesions [5–7]. Secondly, it is possible that UNG2 activity in cells can effectively be depleted by small molecules that disrupt its interaction with PCNA or RPA, and that these molecules would enhance the effectiveness of TS inhibitors. It was reported that PCNA ligands that inhibit its protein interactions can induce replication stress and have additive effects with other DNA damaging agents including cisplatin and UV [56,57]. One caveat is that UNG2 interacts with PCNA and RPA at sites where dozens of other proteins also bind, and targeting these sites would likely affect binding of PCNA and RPA to other proteins in addition to disrupting their interactions with UNG2 [42,58]. Finally, UNG2 was reported to be depleted during treatment with HDAC inhibitors including the clinically-used SAHA and MS275, providing another plausible mechanism to deplete UNG2 activity and sensitive cells to TS inhibitors [59, 60]. Indeed, we confirmed that HDAC inhibitor treatments reduced UNG2 protein levels in HT29 cells with a concomitant reduction in uracil excision activity (Figure S9). Future work will determine whether HDAC inhibitors and TS inhibitors synergize through a UNG2-dependent mechanism or another mode of cytotoxicity [60–62].

We note important aspects of UNG2 biology and TS inhibitor pharmacology that our study did not address. We previously reported that the N-terminal domain of UNG2 interacts with DNA and can target the enzyme to ssDNA-dsDNA junctions independent of PCNA or RPA [16, 63]. It is possible that the interaction of the N-terminal domain with DNA contributed to the localization or retention of UNG2 at the replication fork in the absence of protein interactions. However, the N-terminal domain of UNG2 has a binding affinity for DNA that is at least two orders of magnitude weaker than its affinity for PCNA and RPA, so the role of DNA binding in fork localization may be secondary to the influence of the proteins [16,63]. Our study also focused on the role of nuclear UNG2 and did not directly examine how mitochondrial UNG1 affects the efficacy of TS inhibitors. This is important because our gene editing approach knocked out both isoforms of the enzyme from the parental HT29 cells because they are expressed from the same gene [64]. The finding that re-expression of wild-type UNG2 fully rescued the hypersensitivity of UNG-KO cells to TS inhibitors suggests that UNG1 and mitochondrial base alterations had only a minor impact on TS inhibitor toxicity in our system. Finally, our focus on FdU and pemetrexed found that these drugs become more effective when UNG2’s protein-protein interactions were disrupted. This occurs even as FdU and pemetrexed have different effects on DNA metabolism (Fig. 1A), and pemetrexed also inhibits other folate-dependent enzymes [65]. We did not directly investigate the mechanisms of drug action that led to cell death. Cellular toxicity likely resulted from persistent DNA damage from aberrant or unnatural bases (uracil and 5-fluorouracil), the untimely generation of abasic sites, and/or toxicity associated with cellular thymidine depletion. Previous reports indicate that double-strand breaks resulting from FdU and pemetrexed treatment trigger a caspase-independent apoptotic pathway that is potentiated when UNG2 is not available to mediate DNA repair [5,8–11]. However cell types do not uniformly become hypersensitive to TS inhibitors in the absence of UNG2 which should be considered in understanding the apoptotic mechanism and translating our findings on protein interactions to other cancers [5,8].

In conclusion, we found that the interactions of UNG2 with PCNA and RPA were critical for its enzymatic activity in HT29 colorectal cancer cells. Genetically disrupting the interaction of UNG2 with PCNA or RPA sensitized the cells to the cytotoxic effects of TS inhibitors and promoted DNA damage phenotypes. The key role of UNG2 and its protein-protein interactions in determining sensitivity to TS inhibitors offers several therapeutic strategies to enhance the effectiveness of this class of drugs.

## Supplementary Material

1

## Data Availability

Datasets and image files from this manuscript are available on zenodo at https://zenodo.org/records/17156404 or DOI: 10.5281/zenodo.17156404.

## Appendix A. Supporting information

Supplementary data associated with this article can be found in the online version at doi:10.1016/j.dnarep.2025.103918.
