# Supplementary material for: Efficient activity of uracil DNA glycosylase (UNG2) in proliferating cells requires binding to proliferating cell nuclear antigen (PCNA) and replication protein A (RPA)
Source: DNA Repair (Amst). Author manuscript; Available in PMC 2026 Jun 24. (PMC13292882; doi:10.1016/j.dnarep.2025.103918)
Supplement: 1 [file NIHMS2188797-supplement-1.pdf]

Supplementary Material

Efficient Activity of Uracil DNA Glycosylase (UNG2) in Proliferating Cells Requires Binding to Proliferating Cell Nuclear Antigen (PCNA) and Replication Protein A (RPA)

Rashmi S. Kulkarni,[1,2] Brian P. Weiser[1,2,*]

[1]Department of Cell & Molecular Biology, Rowan-Virtua School of Osteopathic Medicine, Rowan University, Stratford, New Jersey, 08084, United States of America

[2]Department of Cell & Molecular Biology, Rowan-Virtua School of Translational Biomedical Engineering & Sciences, Rowan University, Stratford, New Jersey, 08084, United States of America

*corresponding author email: weiser@rowan.edu

Figure S1. Purification of recombinant UNG2(FF/AA) and UNG2(NR/DG) and the basis for fluorescence anisotropy competition assays. (A) Coomassie-stained SDS-PAGE gel of purified recombinant UNG2(FF/AA) protein. (B) Coomassie-stained SDS-PAGE gel of purified recombinant UNG2(NR/DG) protein. (C) Fluorescence anisotropy assay showing the interaction of Pogo-Ligase peptide with PCNA (see also [1,2]). In the main text, Pogo-Ligase peptide was competed from PCNA with UNG2 variants. (D) Fluorescence anisotropy assay showing the interaction of SMARCAL peptide with the RPA32 winged helix (WH) domain (see also [1]). In the main text, SMARCAL peptide was competed from RPA32-WH with UNG2 variants.

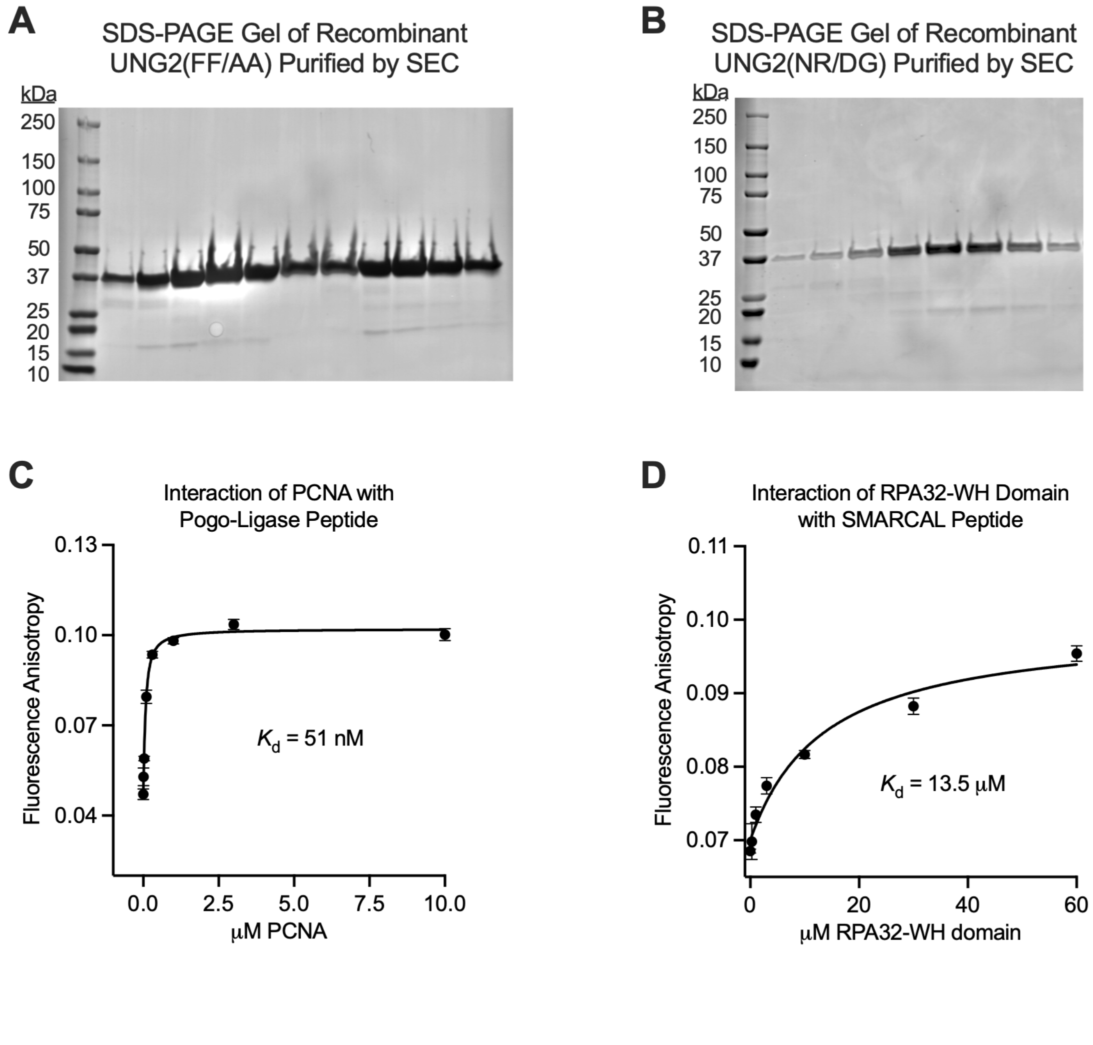

Figure S2. UNG2 expression and uracil excision activity in cell lysates. (A) Western blots of cell lysates from HT29-UNG KO clones 1, 2, and 3; lysate from the parental HT29 cell line is indicated below as "wt". The UNG2 antibody was described in the main text [3–5], and the actin antibody that was used as a loading control was from Sigma-Aldrich (catalog #A1978). (B) Uracil excision assays with cell lysates using 87 bp dsDNA with a U/A base pair as the substrate, as in Figure 2E in the main text. The enzymatic rate constants that are shown below were determined for the reactions that are boxed.

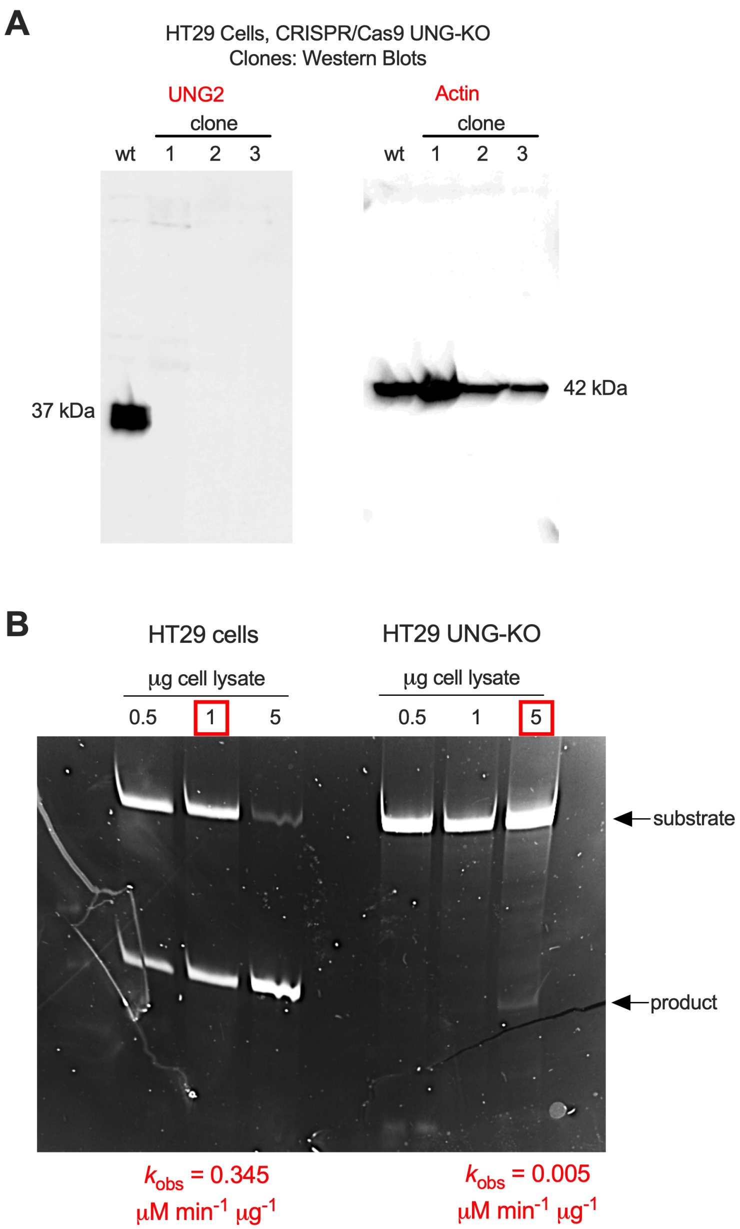

Figure S3. UNG2 activity does not affect the sensitivity of HT29 cells to 5-fluorouracil. HT29 and HT29 UNG-KO cells were treated with 5-fluorouracil, and cell viability was determined with an MTT assay. The IC_{50} represents the dose that inhibited cell viability by 50% as determined by the fit curve, and points represent the mean ± standard error from at least six independent experiments.

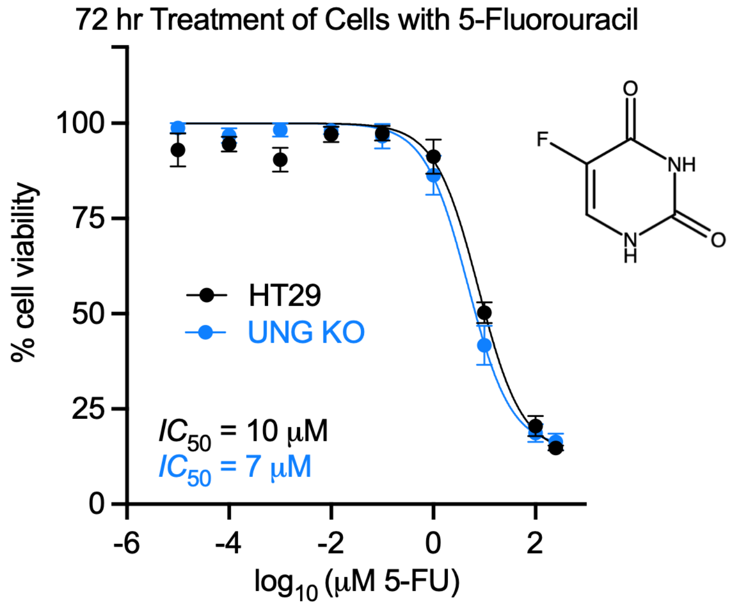

Figure S4. Sensitivity to TS inhibitors was determined by UNG2 and its ability to interact with PCNA and RPA. (A) Sensitivity of HT29 cells, UNG-KO cells, and other cell lines to FdU as determined with a trypan blue dye exclusion assay. The IC_{50} could not be determined for every cell line, so for comparison we reported the percent of viable cells after treatment with 1 μM FdU. (B) Sensitivity of HT29 cells, UNG-KO cells, and other cell lines to pemetrexed as determined with a trypan blue dye exclusion assay. Again, we reported the percent of viable cells after treatment with 1 μM pemetrexed. In panel A and panel B, points represent the mean ± standard error from at least six independent experiments.

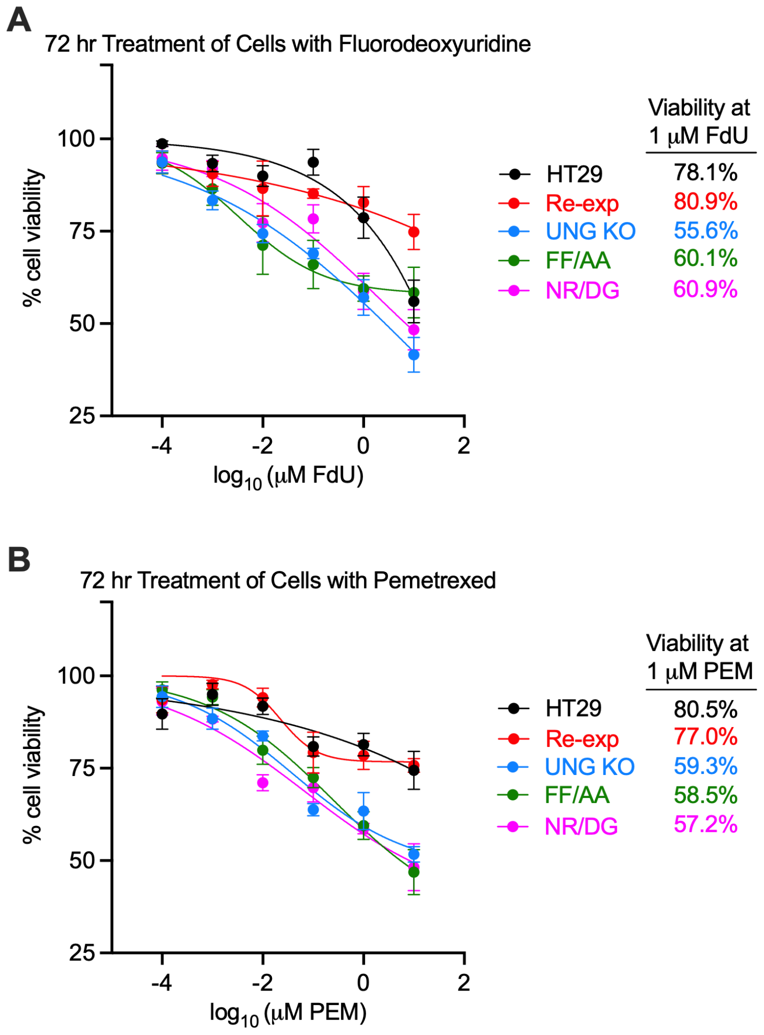

Figure S5. Purified recombinant His-SUMO-UdgX protein and its covalent interaction with uracil-containing DNA. (A) Coomassie-stained SDS-PAGE gel of recombinant His-SUMO-UdgX protein that was purified from *E. coli*. (B) Covalent interaction of His-SUMO-UdgX with 31 nt ssDNA containing a uracil in the center of the strand and a fluorescein end-label [6]. UdgX was incubated with the ssDNA for 30 minutes at 37°C in 10 mM Tris-Cl (pH 8.0), 100 mM NaCl, 0.1mM EDTA, and 1 mM DTT. NaOH was then added to a final concentration of 200 mM, the reactions were diluted three-fold with formamide containing 5 mM EDTA, then the reactions were separated by denaturing urea-TBE PAGE. The reaction with UNG2 was quenched after only three minutes and was performed as a negative control to show the migration of the DNA fragment that results from uracil excision and a reversible protein-DNA interaction.

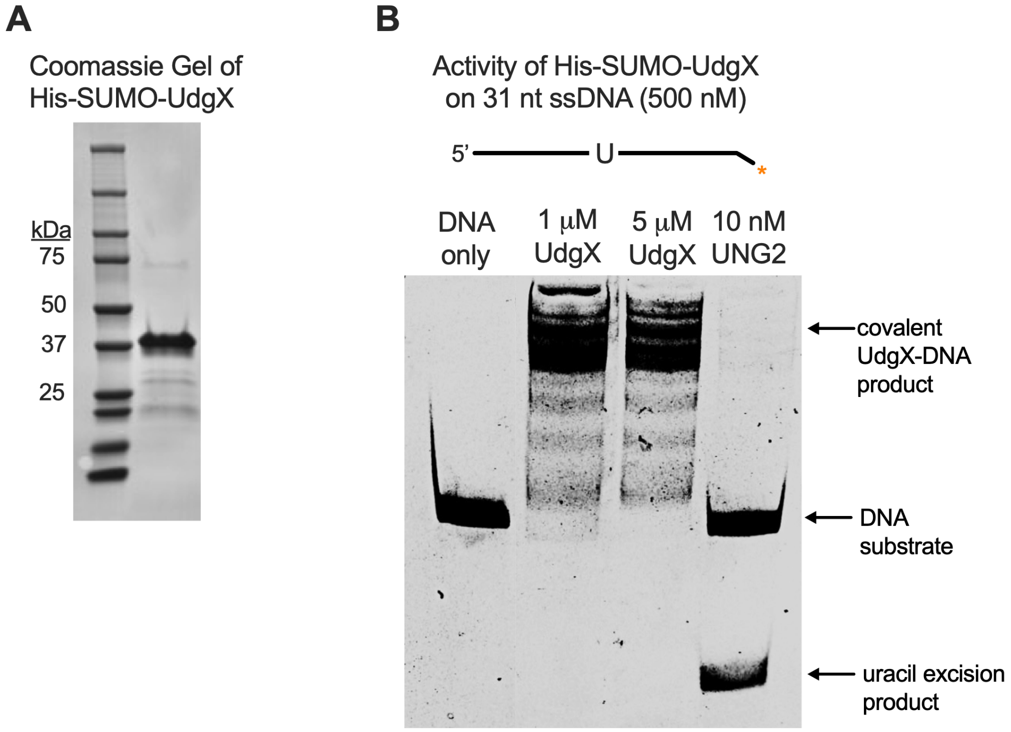

Figure S6. Ability of PCNA, RPA and UNG2 to localize to the replication fork in parental HT29 and UNG-KO cells after 48 hr treatment with DMSO vehicle or 100 nM FdU. Staining of the three proteins was largely diffuse when the cells were treated with DMSO, and discrete foci representing stalled replication forks formed during drug treatment. Quantification of foci localization efficiency for each protein during FdU treatment can be found in Figure 5B of the main text. The authors note that images of the cellular plane were not smoothly stitched together by the microscope software for the DMSO-treated UNG2(NR/DG) control cells.

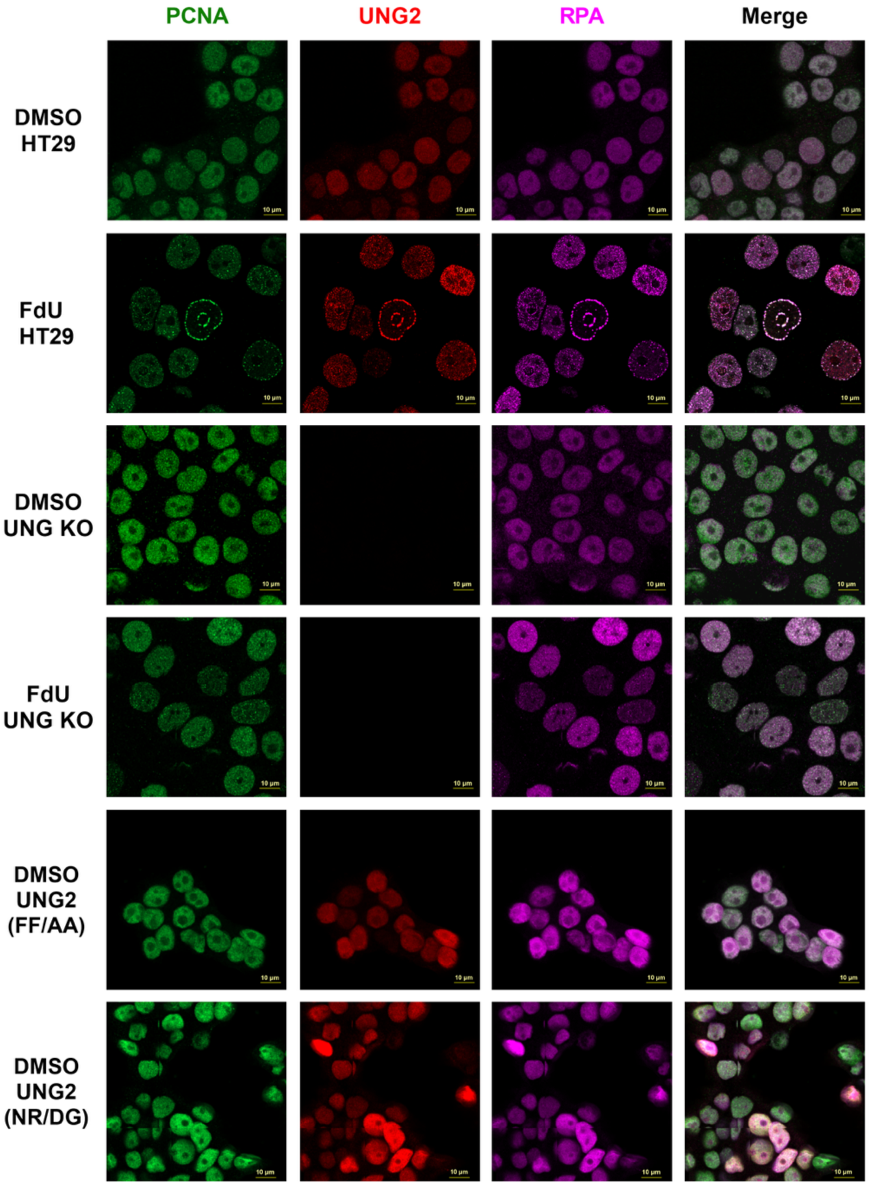

Figure S7. Confocal microscopy images of HT29 UNG-KO cell lines that were re-expressing wild-type UNG2 (Re-exp), UNG2(FF/AA), or UNG2(NR/DG). The cells were treated with DMSO vehicle or 100 nM pemetrexed (PEM) for 48 hr as indicated. RPA, PCNA, and wild-type UNG2 readily co-localized to discrete foci representing stalled replication forks. However, UNG2(FF/AA) and UNG2(NR/DG) sometimes failed to localize to the foci with PCNA and RPA (some examples of this are indicated with the yellow arrows). Quantification of the efficiency of foci localization for each protein during pemetrexed treatment can be found in Figure 5C of the main text.

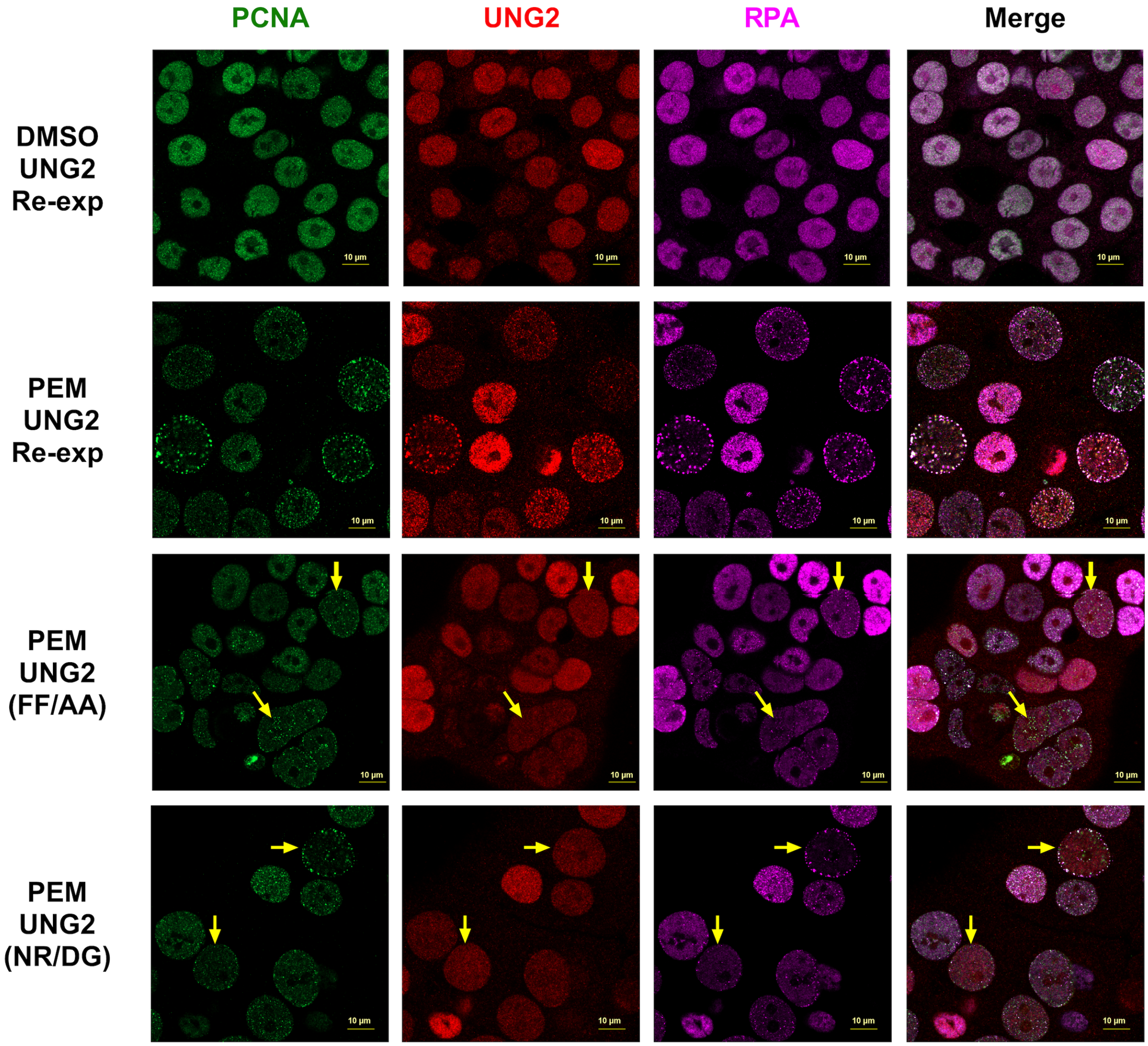

Figure S8. Ability of PCNA, RPA and UNG2 to localize to the replication fork in parental HT29 and UNG-KO cells after 48 hr treatment with DMSO vehicle or 100 nM pemetrexed. Staining of the three proteins was largely diffuse when the cells were treated with DMSO, and discrete foci representing stalled replication forks formed during drug treatment. Quantification of the efficiency of foci localization for each protein during pemetrexed treatment can be found in Figure 5C of the main text.

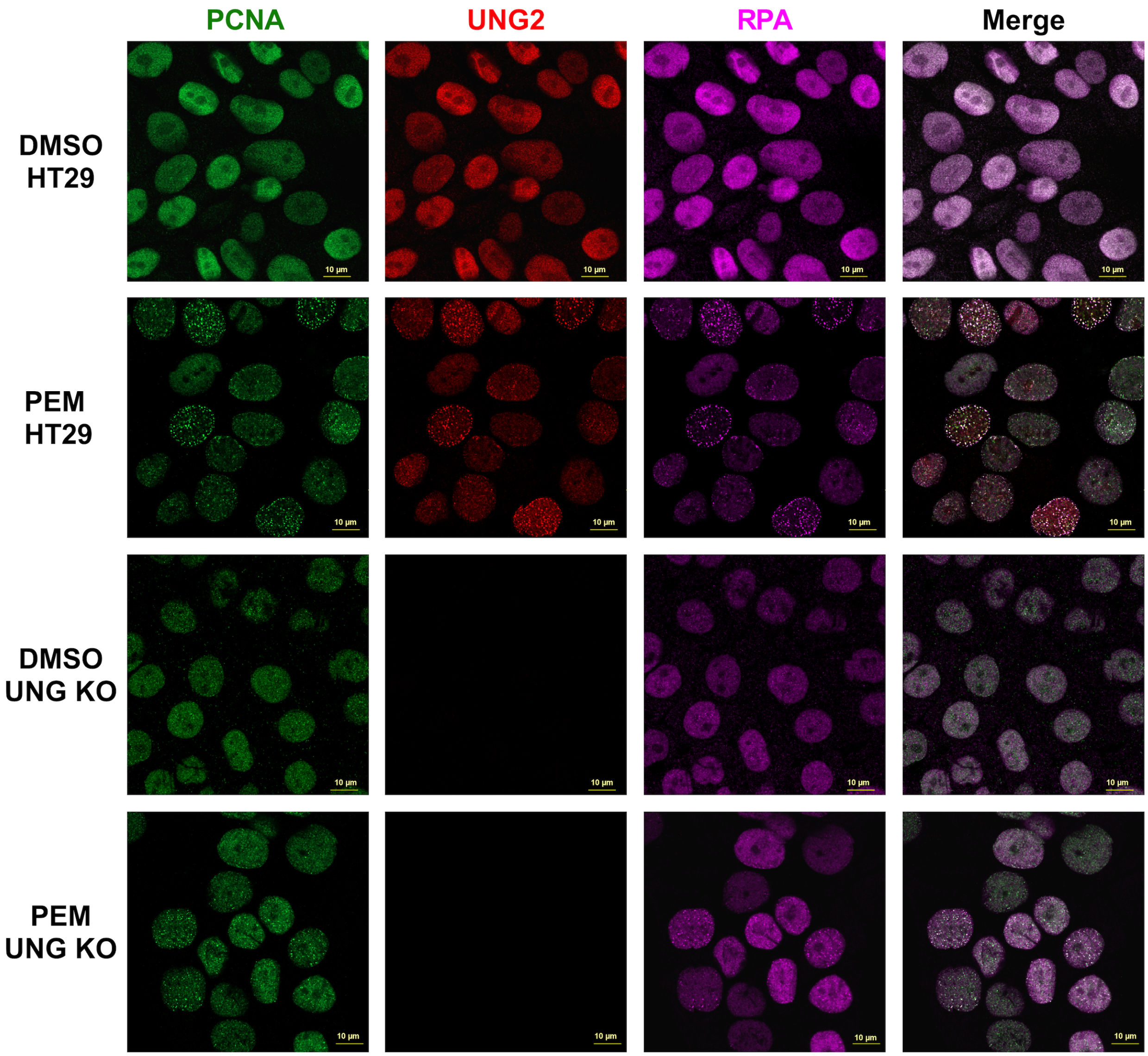

Figure S9. Reduction of UNG2 levels and UNG2 activity in HT29 cells treated with HDAC inhibitors for 24 hours. (A) Representative western blot and the corresponding Coomassie-stained membrane showing UNG2 levels in cell lysates after the indicated treatments with SAHA or MS275. The DMSO concentration in all treatments was 1%. (B) Quantification of western blot data as shown in panel A. The band intensities for the drug-treated groups were normalized to the band intensity of the DMSO-treated control from the same blot. (C) Uracil excision assays that detected reduced UNG2 activity in cell lysates following treatment with HDAC inhibitors. (D) Quantification of uracil excision activity data from assays as shown in panel C. Blots and activity assays were conducted as in Figure 2 of the main text, and all panels have data from three independent experiments.

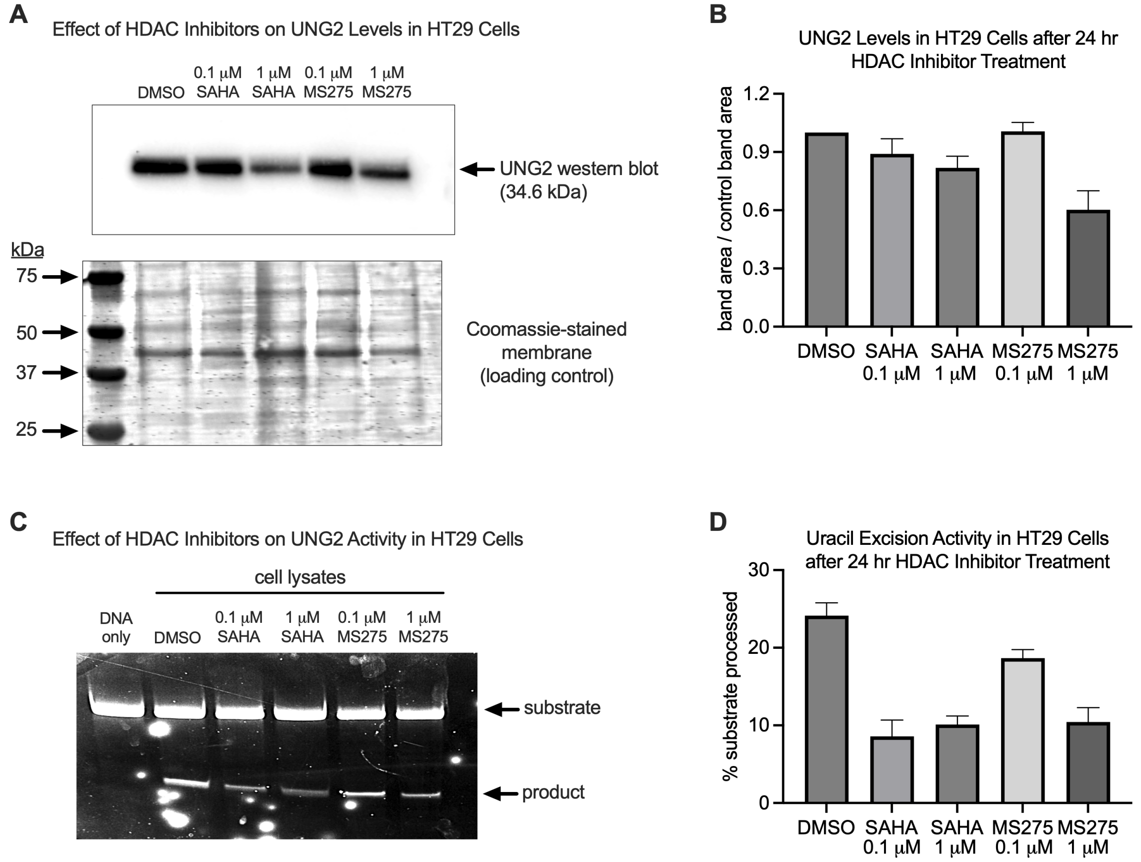

Supplementary References

[1] B.P. Weiser, J.T. Stivers, P.A. Cole, Investigation of N-Terminal Phospho-Regulation of Uracil DNA Glycosylase Using Protein Semisynthesis, Biophys. J. 113 (2017) 393–401. https://doi.org/10.1016/j.bpj.2017.06.016.

[2] S.N. Greenwood, R.G. Belz, B.P. Weiser, A Conserved Mechanism for Hormesis in Molecular Systems, Dose Response 20 (2022) 15593258221109335. https://doi.org/10.1177/15593258221109335.

[3] J.A. Fischer, S. Muller-Weeks, S.J. Caradonna, Fluorodeoxyuridine Modulates Cellular Expression of the DNA Base Excision Repair Enzyme Uracil-DNA Glycosylase, Cancer Res 66 (2006) 8829–8837. https://doi.org/10.1158/0008-5472.CAN-06-0540.

[4] S. Caradonna, S. Muller-Weeks, The nature of enzymes involved in uracil-DNA repair: isoform characteristics of proteins responsible for nuclear and mitochondrial genomic integrity, Curr Protein Pept Sci 2 (2001) 335–347. https://doi.org/10.2174/1389203013381044.

[5] J.A. Fischer, S. Muller-Weeks, S. Caradonna, Proteolytic degradation of the nuclear isoform of uracil-DNA glycosylase occurs during the S phase of the cell cycle, DNA Repair (Amst) 3 (2004) 505–513. https://doi.org/10.1016/j.dnarep.2004.01.012.

[6] S.N. Greenwood, R.S. Kulkarni, M. Mikhail, B.P. Weiser, Replication Protein A Enhances Kinetics of Uracil DNA Glycosylase on ssDNA and Across DNA Junctions: Explored with a DNA Repair Complex Produced with SpyCatcher/SpyTag Ligation, Chembiochem 24 (2023) e202200765. https://doi.org/10.1002/cbic.202200765.
